# Preoperative Prediction of Difficult Laparoscopic Cholecystectomy: Evolution, Validation, and Emerging Machine-Learning Approaches—A Scoping Review

**DOI:** 10.3390/medicina62102042

**Published:** 2026-10-21

**Authors:** Marian Botoncea, Cătălin Dumitru Cosma, Dragoș Călin Molnar, Vlad Olimpiu Butiurca, Cosmin Lucian Nicolescu

**Affiliations:** 1 Faculty of Medicine, George Emil Palade University of Medicine, Pharmacy, Sciences and Technology of Târgu Mureș, 540139 Targu Mures, Romania; marian.botoncea@umfst.ro (M.B.); dragosmolnar2000@yahoo.com (D.C.M.); vlad.butiurca@umfst.ro (V.O.B.); cosmin.nicolescu@umfst.ro (C.L.N.); 2 General Surgery Clinic No. 1, County Emergency Clinical Hospital of Târgu-Mureș, 540136 Targu Mures, Romania

**Keywords:** laparoscopic cholecystectomy, operative difficulty, prediction model, risk score, nomogram, machine learning, radiomics, scoping review

## Abstract

*Background and Objectives*: Preoperative prediction of difficult laparoscopic cholecystectomy (LC) has expanded from point-based scores to regression, nomogram, machine-learning, and quantitative imaging approaches. We examined whether this diversification was accompanied by stronger validation and clinical translation. *Materials and Methods*: Following a registered Open Science Framework protocol, we conducted a scoping review using Joanna Briggs Institute methodology and PRISMA-ScR. PubMed/MEDLINE, Scopus, and Web of Science were searched on 8 August 2026 for English-language publications from 1 January 2010 through 31 July 2026. Eligible studies evaluated structured multivariable tools using exclusively preoperative inputs. Screening and data charting were performed independently and in duplicate. *Results*: Searches identified 893 records; removal of 55 records outside the publication window and 372 duplicates left 466 for screening. Sixty reports were assessed as full text, and 59 were included; 47 (79.7%) were published from 2020 onward. Point-based scores predominated (45/59, 76.3%), followed by nomograms (5/59, 8.5%), regression-based probability models (4/59, 6.8%), machine-learning models (4/59, 6.8%), and one quantitative imaging tool (1/59, 1.7%). Four model-development publications (6.8%) included a geographic external validation component, including one with mixed temporal and geographic validation. Calibration was reported in nine publications (15.3%), decision-curve analysis in seven (11.9%), and SHAP-based explainability in two (3.4%). Two publications evaluated scores during clinical use; no controlled clinical-impact study was identified. *Conclusions*: Evidence of transportability and clinical impact remains limited. Future studies should prioritize standardized prediction targets, complete model specification, calibration, independent validation, and controlled evaluation of model-guided care.

## 1. Introduction

Laparoscopic cholecystectomy (LC) is the standard operative treatment for symptomatic gallstone disease and is performed across elective and emergency settings. Although most procedures are completed safely by a laparoscopic approach, the degree of operative difficulty varies considerably. Acute or recurrent inflammation, fibrosis, adhesions, altered hepatocystic anatomy, impacted stones, and previous biliary interventions may impair exposure and safe anatomical identification. In difficult cases, operative time can increase and surgeons may need subtotal cholecystectomy, conversion to open surgery, or another bailout strategy. Contemporary safety guidance therefore emphasizes achievement of the critical view of safety (CVS), recognition of hazardous anatomy, and timely use of bailout procedures when safe dissection cannot be achieved [1,2].

Preoperative identification of technically demanding cases may help allocate experienced surgeons, plan operating-room time, counsel patients, and anticipate the need for additional resources or alternative operative strategies [3,4,5]. Prediction tools have therefore combined clinical history, inflammatory and biochemical markers, and conventional imaging findings to estimate operative difficulty before surgery [3,4,5]. However, “difficult LC” has no uniform definition. Studies have variously used intraoperative difficulty grades, prolonged operative time, conversion to open surgery, failure to achieve the CVS, subtotal cholecystectomy, or other bailout procedures as prediction targets; these outcomes are related but not interchangeable [1,2,3,5,6,7].

A previous systematic review by Vannucci et al. identified 22 model-development or validation studies through June 2020 and documented nine definitions of operative difficulty, frequent high risk of bias, limited validation in new data, and no clinical-impact evaluations [3]. Since that search period, prediction research has expanded substantially. Large prospective datasets have been used to derive objective preoperative risk scores, evaluations in new populations have increasingly appeared, and recent studies have introduced nomograms, advanced ultrasound techniques, machine learning, radiomics, calibration assessment, decision-curve analysis, and explainability methods [5,7,8,9,10,11,12,13,14,15].

Increasing complexity of the modeling method does not, by itself, demonstrate better clinical decision support. Prediction research should distinguish apparent performance and internal validation from temporal validation and geographic external validation, report calibration as well as discrimination, describe predictor handling and missing data, and evaluate clinically meaningful thresholds and net benefit. TRIPOD+AI and PROBAST+AI provide contemporary frameworks for transparent reporting and appraisal across regression-based and machine-learning prediction models [16,17].

A scoping review approach was selected because the primary objective was to map the breadth and methodological evolution of preoperative prediction tools rather than to estimate a pooled measure of predictive performance or identify a single superior model. The available literature encompasses heterogeneous prediction targets, clinical populations, predictor modalities, modeling approaches, and validation strategies, limiting the appropriateness of direct quantitative comparisons. Following JBI methodology, a scoping review allows these characteristics to be systematically mapped while identifying important evidence gaps, particularly regarding calibration, external validation, model reproducibility, and clinical implementation.

Against this background, this scoping review aimed to map structured preoperative prediction tools for difficult laparoscopic cholecystectomy published between 2010 and July 2026, with particular emphasis on their clinical applicability and methodological validation. Specifically, we examined which clinical, laboratory, and imaging inputs are commonly used, which models have undergone independent validation, how readily their predictions can be generated in routine practice, and whether their implementation has been shown to influence perioperative decision-making or patient outcomes. By addressing these questions, the review sought to distinguish methodological development from practical clinical readiness and identify priorities for future research.

## 2. Materials and Methods

### 2.1. Design, Reporting, and Protocol

This scoping review was conducted in accordance with the Joanna Briggs Institute (JBI) methodology for scoping reviews and reported according to the PRISMA Extension for Scoping Reviews (PRISMA-ScR) [18,19]. The completed PRISMA-ScR checklist is available in the Supplementary Materials (PRISMA-ScR Checklist). Eligibility was structured using the Population–Concept–Context framework. The review protocol was registered on the Open Science Framework (OSF) (doi: 10.17605/OSF.IO/GQYH8) and prespecified the review period, eligibility criteria, information sources, data-charting domains, and narrative and graphical synthesis plan.

During review conduct, a protocol amendment was documented on OSF to remove the originally planned backward citation-searching step. Consequently, study identification was restricted to the three prespecified electronic databases: PubMed/MEDLINE, Scopus, and Web of Science Core Collection. No other substantive changes were made to the eligibility framework, data-charting domains, or synthesis plan.

### 2.2. Eligibility Criteria

The population comprised adults undergoing or scheduled to undergo LC for benign gallbladder disease, including elective, emergency, and interval procedures following preoperative gallbladder drainage. Mixed populations were eligible when data for adult patients undergoing LC were separately identifiable or represented the predominant study population. Only full-text primary studies published in English were eligible.

The central concept was a structured multivariable prediction model or score whose required inputs consisted exclusively of information available before the start of surgery. Eligible methodological roles included development of a new preoperative score or prediction model; internal resampling, internal split-sample, temporal, or geographic external validation; independent validation of an existing published model or score; model comparison, updating, recalibration, modification, or extension; and implementation or impact evaluation. Among studies retrieved through the prespecified difficult-LC search framework, models predicting conversion to open surgery, failure to achieve the CVS, subtotal cholecystectomy, or another bailout procedure were eligible when the endpoint was explicitly conceptualized as an indicator, surrogate, or component of operative difficulty.

Studies were excluded if they evaluated only isolated predictors; reported multivariable associations without deriving or formally evaluating a reproducible prediction tool; applied an established score locally without substantive validation, comparison, recalibration, updating, or methodological advancement; required any predictor obtained intraoperatively, postoperatively, or through histopathological examination; predicted postoperative outcomes without an operative-difficulty target; or were reviews, meta-analyses, editorials, letters, protocols, case reports, or conference abstracts.

### 2.3. Definition of Difficult Laparoscopic Cholecystectomy

Throughout this review, “difficult LC” is used as an umbrella term encompassing study-defined manifestations or proxies of difficulty during laparoscopic cholecystectomy. “Operative difficulty” refers to the technical demands of the procedure, while specific prediction targets are described using their original operational definitions. The term “surgical difficulty” is retained only when reproducing terminology from the original publications or search strategies.

We did not impose a uniform operational definition of difficult LC because this heterogeneity was itself a focus of the review. Eligible definitions included validated or investigator-defined intraoperative difficulty grades, difficult dissection, severe adhesions or inflammatory changes, impaired anatomical identification, failure to achieve the CVS, prolonged operative time, conversion to open surgery, subtotal cholecystectomy, bailout procedures, and composite endpoints. These outcomes were treated as related but non-interchangeable manifestations or proxies of difficult LC. Each study’s operational definition was retained rather than retrospectively reclassified.

### 2.4. Information Sources and Search Strategy

PubMed/MEDLINE, Scopus, and Web of Science Core Collection were searched on 8 August 2026. The prespecified publication window was 1 January 2010 through 31 July 2026, and searches were restricted to English-language publications using database-specific filters where available. The search strategy combined terms related to laparoscopic cholecystectomy, operative difficulty or conversion, and prediction-model development. Database-specific syntax was adapted as follows.

The selection of PubMed/MEDLINE, Scopus, and Web of Science Core Collection was established a priori in the registered OSF protocol. These databases were chosen to provide broad coverage of biomedical and multidisciplinary research relevant to preoperative prediction models. To maintain consistency with the prespecified search methodology, no additional databases were introduced during the review process.

PubMed/MEDLINE: 

(“Cholecystectomy, Laparoscopic” [Mesh] OR “laparoscopic cholecystectomy” [Title/Abstract]) AND (“difficult laparoscopic cholecystectomy” [Title/Abstract] OR “difficult cholecystectomy” [Title/Abstract] OR “operative difficulty” [Title/Abstract] OR “surgical difficulty” [Title/Abstract] OR “difficulty prediction” [Title/Abstract] OR “conversion to open” [Title/Abstract]) AND (predict*[Title/Abstract] OR score*[Title/Abstract] OR “scoring system” [Title/Abstract] OR “prediction model” [Title/Abstract] OR “predictive model” [Title/Abstract] OR nomogram*[Title/Abstract] OR “machine learning” [Title/Abstract] OR “artificial intelligence” [Title/Abstract] OR radiomic*[Title/Abstract]) AND (“2010/01/01” [Date—Publication] : “2026/07/31” [Date—Publication]).

Scopus:

TITLE-ABS (“laparoscopic cholecystectomy”) AND TITLE-ABS (“difficult laparoscopic cholecystectomy” OR “difficult cholecystectomy” OR “operative difficulty” OR “surgical difficulty” OR “difficulty prediction” OR “conversion to open”) AND TITLE-ABS (predict* OR score* OR “scoring system” OR “prediction model” OR “predictive model” OR nomogram* OR “machine learning” OR “artificial intelligence” OR radiomic*) AND PUBYEAR > 2009 AND PUBYEAR < 2027.

Web of Science Core Collection:

(TI = (“laparoscopic cholecystectomy”) OR AB = (“laparoscopic cholecystectomy”)) AND (TI = (“difficult laparoscopic cholecystectomy” OR “difficult cholecystectomy” OR “operative difficulty” OR “surgical difficulty” OR “difficulty prediction” OR “conversion to open”) OR AB = (“difficult laparoscopic cholecystectomy” OR “difficult cholecystectomy” OR “operative difficulty” OR “surgical difficulty” OR “difficulty prediction” OR “conversion to open”)) AND (TI = (predict* OR score* OR “scoring system” OR “prediction model” OR “predictive model” OR nomogram* OR “machine learning” OR “artificial intelligence” OR radiomic*) OR AB = (predict* OR score* OR “scoring system” OR “prediction model” OR “predictive model” OR nomogram* OR “machine learning” OR “artificial intelligence” OR radiomic*)).

The search strategy was intentionally centered on the overarching concepts of difficult laparoscopic cholecystectomy, operative or surgical difficulty, and conversion to open surgery. Safety-oriented endpoints such as failure to achieve the critical view of safety, subtotal cholecystectomy, or bailout procedures were considered eligible when they were explicitly framed by the original study as manifestations, surrogates, or components of operative difficulty and were retrieved through the prespecified search strategy. These endpoints were not searched as independent concepts.

The searches retrieved 148 records from PubMed/MEDLINE, 371 from Scopus, and 374 from Web of Science, yielding 893 records before cleaning. Because the Scopus and Web of Science searches included the full 2026 publication year at the database-filter level, the prespecified cutoff of 31 July 2026 was applied during dataset cleaning. Overall, 55 records falling outside the prespecified publication window were removed before title/abstract screening. The remaining records were imported into Zotero (version 9.0.6, Corporation for Digital Scholarship, Vienna, VA, USA) for reference management and deduplication. Deduplication was then performed hierarchically using DOI, PMID, and normalized title, with stable identifiers prioritized and conflicting records manually adjudicated. No additional citation searching, hand-searching, or grey-literature searches were performed.

### 2.5. Study Selection

Study selection was performed independently and in duplicate by two reviewers (C.D.C. and V.O.B.). Both reviewers assessed titles and abstracts against the prespecified eligibility criteria and subsequently evaluated the retrieved full-text reports. Potentially eligible or uncertain records were advanced to full-text retrieval. Any uncertainties or disagreements arising during study selection were resolved by discussion with a third reviewer (C.L.N.).

Full texts were sought through publisher websites, bibliographic databases, institutional access, and publicly available repositories. In accordance with the prespecified OSF protocol, study authors were not contacted to obtain unavailable reports. Reports that could not be retrieved through these sources were classified as reports not retrieved and did not undergo full-text eligibility assessment. Full-text assessment focused on three key eligibility boundaries: whether the study evaluated a reproducible structured multivariable prediction tool; whether all inputs required to calculate the tool were available before the start of surgery; and, for validation, comparison, updating, or extension studies, whether the evaluation represented a substantive methodological assessment rather than simple local reapplication. One primary reason for exclusion was assigned to each report excluded after full-text assessment.

### 2.6. Data Charting, Prediction-Tool Classification, and Validation Strategy

The unit of analysis for descriptive synthesis was the eligible full-text publication/report. Each included publication contributed one study-level entry to the evidence map; accordingly, the final dataset comprised 59 included publications/reports and 59 corresponding analytic study entries. The term “study” is used subsequently for readability to refer to these analytic entries and should not be interpreted as implying that all underlying patient cohorts were necessarily independent or non-overlapping. All study-level percentages reported in the synthesis therefore use the 59 included publications as the denominator.

Data charting was performed independently and in duplicate by C.D.C. and V.O.B. using the prespecified extraction framework, with uncertainties or discordant entries resolved by discussion with C.L.N. Charted domains included bibliographic characteristics, study design and setting, sample size, clinical context, definition of difficult LC, predictor modality, model role, predominant prediction-tool category, model-development methods, predictive performance, validation strategy, calibration, decision-curve analysis, explainability, and clinical translation.

For publications involving machine-learning approaches, additional methodological details were charted regarding class imbalance handling, feature selection, hyperparameter tuning, cross-validation, and reported measures to prevent data leakage. Unreported details were recorded as NR. Where data separation was described but the restriction of preprocessing or feature selection to training data was unclear, this uncertainty was explicitly noted. These methodological details are presented in Supplementary Table S1, Panel D.

For descriptive synthesis, each publication was assigned to one mutually exclusive predominant prediction-tool category according to the form and analytical framework of the principal tool presented in the study: (1) point-based prediction score; (2) regression-based prediction model presented primarily as an equation or probability model; (3) regression-derived model presented primarily as a nomogram; (4) machine-learning model; or (5) imaging-derived quantitative prediction tool not primarily represented by the preceding categories. This taxonomy was intended as a pragmatic description of the principal tool presented by each study rather than as a hierarchy of mutually exclusive statistical methodologies. Because a nomogram represents a model presentation format rather than a distinct statistical method, nomogram-based tools were identified separately only when the nomogram constituted the principal clinically presented prediction instrument. Predictor modality—including clinical/history variables, laboratory or inflammatory markers, conventional ultrasonography, cross-sectional imaging, advanced ultrasound, radiomics or other quantitative imaging features, and multimodal combinations—was charted independently and was not used to define the prediction-tool category. Model role (development, validation, comparison, updating, or implementation) was also recorded separately and did not determine the predominant prediction-tool category.

Validation strategy was classified according to the relationship between the development and evaluation data rather than the terminology used by the original authors. Categories comprised apparent performance; internal resampling validation, including bootstrap or cross-validation; internal split-sample validation; temporal validation; geographic external validation; and independent validation of an existing published model or score in a new cohort. Random division of a single-source cohort into development and validation subsets was classified as internal split-sample validation rather than external validation. Prospective implementation or impact evaluation was recorded separately and was not considered equivalent to external validation. For the quantitative summary of validation maturity, each publication was classified according to the most advanced validation strategy demonstrated.

### 2.7. Synthesis

Because the included studies used non-equivalent prediction targets, populations, thresholds, and validation procedures, quantitative pooling and indirect ranking of model performance were not considered appropriate. Counts and percentages were used to summarize publication timing, predominant prediction-tool categories, validation strategies, reporting of calibration, decision-curve analysis, and formal explainability, as well as major predictor modalities and prediction-target features. Predictor modalities and prediction-target features were summarized as non-mutually exclusive domains because individual studies could incorporate multiple preoperative information sources or more than one manifestation of operative difficulty. Narrative synthesis focused on prediction targets, predictor modalities, validation maturity, discrimination and calibration, clinical-utility assessment, explainability, and evolution in prediction-tool design and presentation, from predominantly point-based scores toward a broader range of regression-based models, regression-derived nomograms, machine-learning models, and quantitative imaging tools. To account for unequal publication periods, annualized publication counts were calculated by dividing the number of included publications in each period by its duration in years.

Consistent with the descriptive and mapping objectives of a scoping review, no formal study-level risk-of-bias, applicability, or certainty-of-evidence assessment was undertaken. Methodological features relevant to prediction-model development, validation, reporting, reproducibility, and clinical translation were instead mapped descriptively across the included publications. Accordingly, terms used in this review such as methodological maturity, validation strength, reporting completeness, transportability, and clinical readiness refer to descriptive characteristics of the evidence base and should not be interpreted as formal PROBAST+AI risk-of-bias or applicability judgments.

Generative AI was not used for study eligibility decisions, primary data extraction, or the generation of study data. All quantitative summaries, tables, and figures were based exclusively on author-verified study-level data and were reviewed and verified by the authors.

## 3. Results

### 3.1. Study Identification, Screening, and Inclusion

Database searches identified 893 records. Fifty-five records falling outside the registered publication window were removed, followed by 372 duplicates, leaving 466 records for title/abstract screening. Of these, 402 were excluded and 64 reports were sought for retrieval. Sixty reports were retrieved, whereas four could not be obtained through the prespecified retrieval sources and were classified as reports not retrieved. Details of these four reports are provided in Supplementary Table S2.

**Figure 1:**
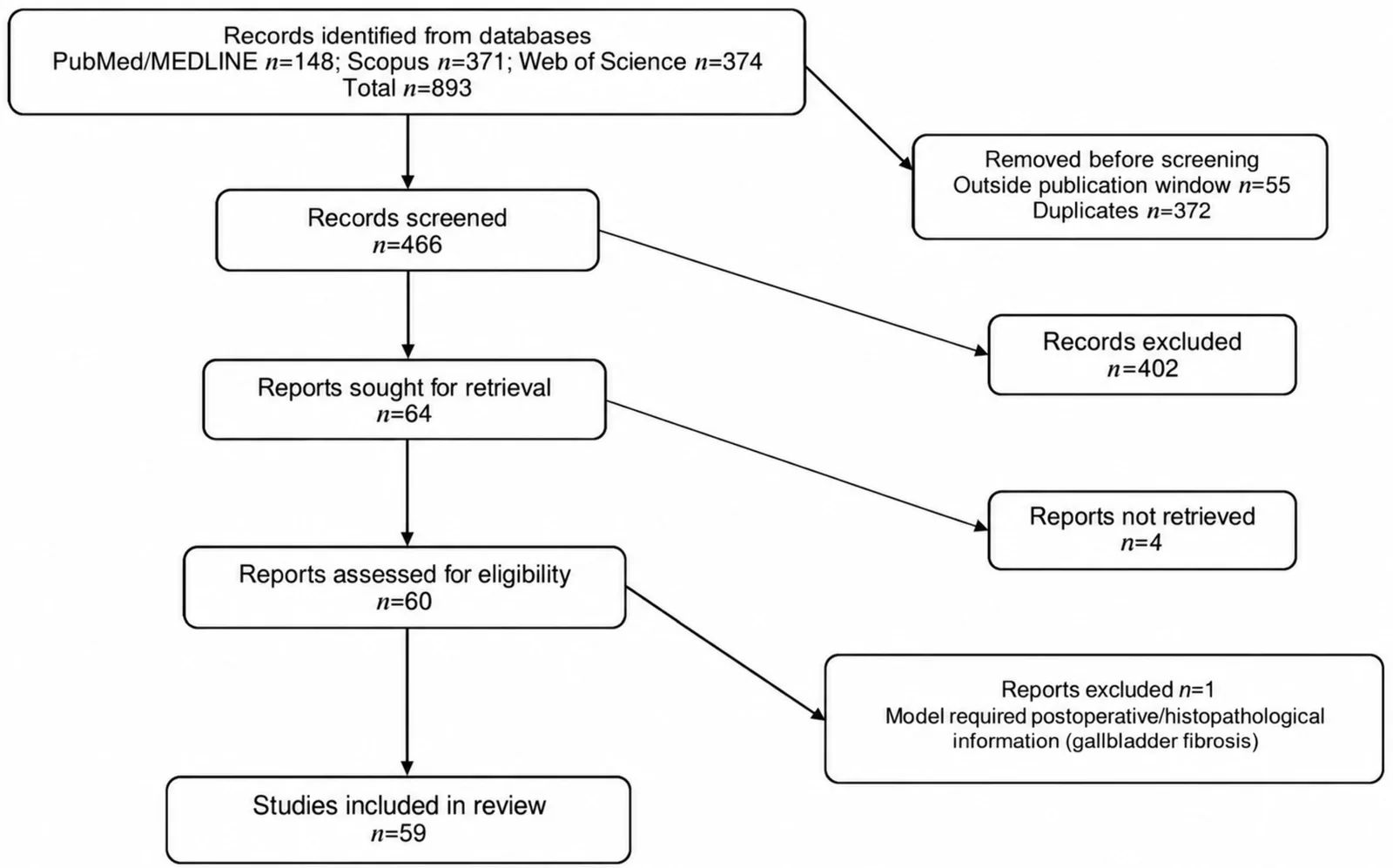
PRISMA-ScR flow diagram of the study selection process.

The 60 retrieved reports underwent full-text eligibility assessment. One report was excluded because the final prediction model required postoperative histopathological information (gallbladder fibrosis) and therefore could not be calculated entirely before the start of surgery. Further details regarding the excluded article are provided in Supplementary Table S2. Fifty-nine eligible full-text publications/reports were included in the final evidence synthesis, each contributing one analytic study entry (*n* = 59) (Figure 1) [4,5,6,7,8,9,10,11,12,13,14,15,20,21,22,23,24,25,26,27,28,29,30,31,32,33,34,35,36,37,38,39,40,41,42,43,44,45,46,47,48,49,50,51,52,53,54,55,56,57,58,59,60,61,62,63,64,65,66].

### 3.2. Publication Timeline

The 59 included studies were published between January 2010 and July 2026. Three studies (5.1%) were published in 2010–2014, nine (15.3%) in 2015–2019, 25 (42.4%) in 2020–2024, and 22 (37.3%) in 2025–31 July 2026. Overall, 47 of 59 studies (79.7%) were published from 2020 onward, demonstrating a substantial recent expansion of research on preoperative prediction of difficult LC (Table 1).

After accounting for unequal observation windows, publication rates increased from 0.6 publications/year in 2010–2014 to 1.8 in 2015–2019, 5.0 in 2020–2024, and 13.9 in January 2025–July 2026 (Table 1).

### 3.3. Predominant Prediction-Tool Category

**Figure 2:**
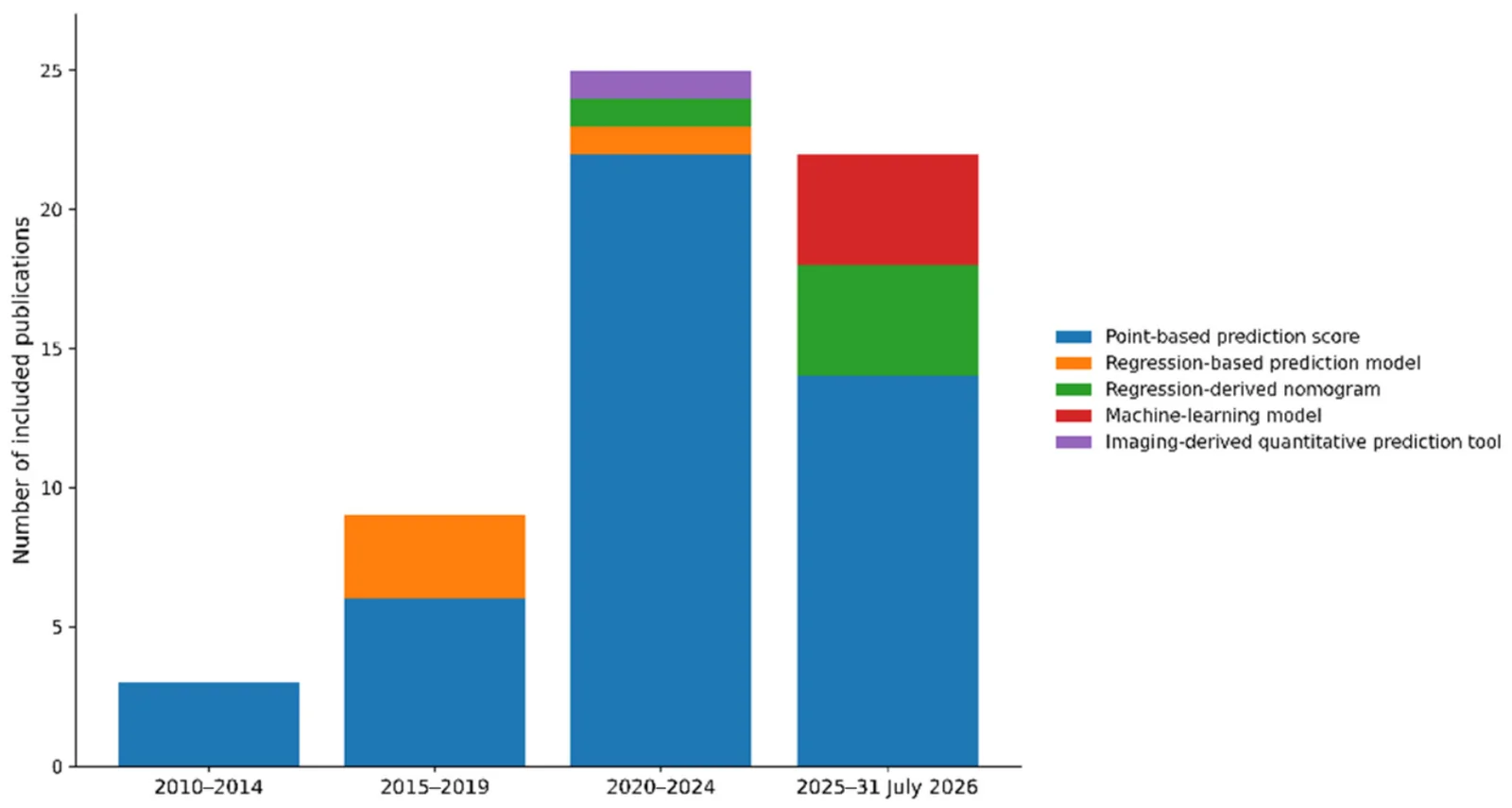
Temporal distribution of predominant prediction-tool categories among the 59 included publications. Categories describe the principal form or analytical framework of the prediction tool presented in each publication; predictor modality and model role were charted separately. Bars represent unadjusted publication counts across periods of unequal duration; corresponding annualized publication rates are provided in Table 1.

Using the pragmatic prediction-tool taxonomy defined above, point-based prediction scores were the predominant category, accounting for 45 of 59 studies (76.3%). Five studies (8.5%) presented regression-derived models primarily as nomograms, four (6.8%) presented regression-based probability models without a nomogram as the principal tool, four (6.8%) used machine-learning models, and one (1.7%) evaluated an imaging-derived quantitative prediction tool. These categories describe the principal form in which the prediction tool was developed or presented and should not be interpreted as mutually exclusive statistical methodologies. Predictor modality and model role were analyzed separately (Table 2), while their temporal evolution across publication periods is illustrated in Figure 2.

### 3.4. Prediction Targets, Definitions of Difficulty, and Predictor Modalities

Prediction targets remained heterogeneous throughout the review period. Many studies predicted a generic binary or graded construct of difficult LC, whereas others focused on prolonged operative time, conversion to open surgery, objective intraoperative difficulty grades, failure to achieve the CVS, or the anticipated need for bailout surgery. These outcomes were considered related but non-interchangeable manifestations or proxies of difficult LC. For example, the large CholeS-based model by Nassar et al. used an objective intraoperative grading system and evaluated a preoperative score with internal and geographic external validation for grade 3–5 difficulty [5], whereas other models focused specifically on conversion to open surgery [13,24,25,28] or bailout surgery [7].

Predictor modalities were also heterogeneous. Conventional models most commonly combined demographic and clinical-history variables with inflammatory or biochemical markers and ultrasonographic findings. More recent approaches increasingly incorporated CT-derived variables, systemic inflammatory indices, advanced feature-selection methods, and quantitative imaging or radiomics-derived features.

Quantitative mapping confirmed the breadth of this heterogeneity (Table 3). Clinical/history variables were incorporated in 50 studies (84.7%), conventional ultrasonography or sonography in 44 (74.6%), and laboratory or inflammatory markers in 19 (32.2%). CT or other explicitly cross-sectional imaging was used in five studies (8.5%), whereas advanced ultrasonography and radiomics were each represented in one study (1.7%). Overall, 51 studies (86.4%) combined more than one predictor modality.

This methodological expansion increased the range of available prediction strategies but also limited direct comparability across studies when prediction targets, patient populations, predictor definitions, and decision thresholds differed.

Prediction targets were similarly diverse. Generic or ordinal difficult-LC constructs remained the largest single descriptive group (26/59, 44.1%), while conversion to open surgery was explicitly incorporated as a target in 13 studies (22.0%). Objective intraoperative grading systems such as Nassar or Parkland grades were used in six studies (10.2%), operative-time-based definitions in five (8.5%), composite difficult-LC endpoints in six (10.2%), CVS-related endpoints in two (3.4%), and bailout procedures in two (3.4%). Because some studies incorporated more than one manifestation of operative difficulty, these target features were not treated as mutually exclusive.

### 3.5. Validation and Methodological Maturity

Validation was standardized according to the relationship between the data used to develop or modify a tool and those used to evaluate it. Apparent performance, internal validation, temporal validation, and geographic external validation were distinguished from independent evaluation of an existing published model or score. Prospective recruitment was recorded as a study-design characteristic. Validation was assessed for the specific tool evaluated, because some publications combined development of a new model with evaluation of an established comparator. Study-level details are provided in Supplementary Table S1.

Seven publications (11.9%) used an internal holdout sample [7,10,12,15,24,52,53]. These included random splits of single-center or pooled multicenter datasets and the small sequential training/test split reported by Agrawal et al. [52]. Multicenter recruitment alone did not establish external validation: Sutcliffe and Suzuki randomly divided pooled patient data, while Sun evaluated a holdout sample from the same center [7,10,24]. Three further publications (5.1%) used resampling as their principal internal validation, without a separate holdout or external cohort: Bourgouin, Moreno Delgado, and Şenocak [4,59,61]. Some studies with holdout or external cohorts also used resampling during model development.

Four publications (6.8%) combined development of a new tool with evaluation in a geographically separate or partly geographically separate cohort [5,13,14,35]. Nassar evaluated the score in a separate reference cohort, Wu used seven external centers, and Yang used two hospitals excluded from development [5,13,14]. Matsumoto combined later patients from the development hospital with patients from a second hospital; this represented mixed temporal and geographic validation [35]. Independent geographic validation of an existing tool was also demonstrated by Hu, who evaluated the unchanged Cairns Prediction Model in a different hospital [28].

Established scores were frequently assessed in new institutional populations, including the Randhawa-based evaluations by Gupta, Kumar, Reddy, and Paul [22,32,37,38]. These studies evaluated previously published tools rather than developing new models, although some selected thresholds within the evaluation cohort. Vyas, Goyal, and Stoica evaluated established comparators while proposing new scores or regression models whose performance remained apparent [20,33,48]. Kapoor likewise assessed the new adhesion-detection system in the same cohort used for its diagnostic comparison [8]. Changes to predictors, weights, or thresholds were recorded explicitly; evaluations of locally adapted rules, such as those reported by Wibowo, Morsy, Ahmed, and Kumar, did not establish transportability of the original unchanged score [34,42,57,63].

Calibration assessment for a developed prediction model was reported in nine publications (15.3%) [9,10,12,13,14,15,39,53,61]. Reporting ranged from a Hosmer–Lemeshow test to calibration curves, with calibration slopes or quantitative errors available in some studies. A goodness-of-fit test for a secondary association model was not attributed to the evaluated clinical score. Decision-curve analysis was reported in seven publications (11.9%) [9,10,13,14,15,53,61], and formal explainability analysis in two (3.4%), both using SHAP [10,14]. These features were concentrated in recent regression, nomogram, and machine-learning studies and were recorded separately from validation in independent data.

Evidence of clinical implementation remained limited. Zhang prospectively evaluated a score-guided management pathway, and Ayantunde evaluated the Traffic Light score during routine theatre booking [6,65]. These studies documented clinical use without a controlled comparison establishing the effect of model-guided care on patient outcomes. Decision-curve analysis and the availability of a calculator were considered separately from observed implementation or clinical impact.

### 3.6. Recent Nomogram, Quantitative Imaging, and Machine-Learning Approaches

Quantitative imaging and machine-learning approaches remained a minority of the evidence base and were concentrated in recent years. Kapoor et al. [8] evaluated an acoustic-radiation-force-impulse virtual-touch-imaging adhesion-detection system in a prospective same-cohort diagnostic comparison. The reported AUC for the adhesion-detection system was inconsistent across the publication, with a value of 1.00 in the Abstract and approximately 0.91 in the Results/figure, compared with approximately 0.63 for the conventional comparator; no independent validation was performed [8]. In 2025, a CT radiomics–clinical machine-learning study evaluated six algorithms in 2055 patients. The final random-forest model achieved an AUC of 0.938 in the development data and 0.874 in an internal split-sample validation set, with calibration, decision-curve analysis, and SHAP-based interpretation also reported [10]. A separate machine-learning-based nomogram achieved AUCs of 0.774 and 0.863 in the development and internal split-sample validation datasets, respectively [53].

Recent nomogram-based studies also extended prediction toward safety-oriented endpoints. Suzuki et al. used a pooled five-institution cohort of 1898 patients, randomly divided into development and validation subsets, to develop a bailout-surgery nomogram; AUCs were 0.788 and 0.769, respectively, representing internal split-sample rather than geographic external validation [7]. An eight-center conversion nomogram underwent geographic external validation, with AUCs of 0.868 in development and 0.833 in the external cohort, alongside calibration and decision-curve analysis [13]. In 2026, a three-center machine-learning study following percutaneous transhepatic gallbladder drainage reported AUCs of approximately 0.881 in development and 0.868 in a geographically external cohort, together with cross-validation, calibration, decision-curve analysis, SHAP-based interpretation, and an online calculator [14]. Utsumi et al. used an internal split-sample design for an inflammation-based nomogram, reporting AUCs of 0.806 in development and 0.760 in internal validation data [15].

### 3.7. Clinical Applicability and Validation of Selected Prediction Tools

To provide a clinically oriented synthesis, selected prediction tools were compared according to their preoperative data requirements, model format, validation maturity, and documented clinical application (Table 4). These examples illustrate differences in practical feasibility and readiness for perioperative decision support rather than a ranking of predictive performance. Complete study-level characteristics for all 59 included publications are presented in Supplementary Table S1.

### 3.8. Clinical Translation

The clinical applicability of preoperative prediction tools varied according to their input requirements, model complexity, and validation status. Conventional point-based scores using routinely available clinical and ultrasonographic information may be more readily incorporated into preoperative assessment, whereas radiomics-based and machine-learning approaches may require additional imaging analysis, computational infrastructure, or dedicated calculators. However, practical feasibility alone does not establish clinical effectiveness.

Evidence of actual implementation remained limited. Zhang et al. [6] prospectively evaluated a score-guided management pathway incorporating risk-adapted surgical allocation, while Ayantunde et al. [65] assessed the Traffic Light score during routine theatre booking to support operative scheduling and surgeon review. These studies illustrate how prediction tools may influence perioperative workflows, but neither provides controlled evidence of improved patient outcomes. No controlled clinical-impact study comparing model-guided management with usual care was identified.

## 4. Discussion

### 4.1. Principal Findings

This scoping review maps a rapidly expanding literature on structured preoperative prediction of difficult LC. Across the 59 included studies, four findings are particularly important. First, research activity has accelerated substantially, with 47 of 59 studies (79.7%) published from 2020 onward. Second, point-based prediction scores remain dominant (45/59, 76.3%), despite the recent emergence of regression-based probability models, regression-derived nomograms, machine-learning models, and imaging-derived quantitative prediction tools [7,8,9,10,13,14,15,53].

Third, methodological diversification has progressed more rapidly than evidence of model transportability and clinical usefulness. Although discrimination was commonly reported, calibration was assessed in only nine publications (15.3%) [9,10,12,13,14,15,39,53,61], decision-curve analysis in seven (11.9%) [9,10,13,14,15,53,61], and SHAP-based explainability in two (3.4%) [10,14]. Although two publications evaluated prediction tools during clinical use [6,65], no controlled clinical-impact study was identified. The limited evaluation beyond discrimination represents a central methodological gap: a high AUC alone does not establish reliable individual risk estimates, beneficial clinical decisions, or improved surgical outcomes [16,17]. These findings support prioritizing calibration, independent validation, and prospective evaluation of model-guided care.

Fourth, the publication/report was the unit of descriptive analysis, and the 59 included publications do not necessarily represent 59 independent patient cohorts. Confirmed or possible cohort overlap was identified between studies using the CholeS cohort [5,24] and between publications by Ramírez-Giraldo et al. [36,45], as documented in Supplementary Table S1. Such overlap may lead to repeated representation of patient populations in the evidence map. Publication-level counts and percentages should therefore be interpreted as measures of research activity rather than independent replication.

The review further highlights a conceptual distinction that is essential for interpreting this literature: identification of independent risk factors does not, by itself, constitute development or validation of a prediction model. This distinction is consistent with contemporary prediction-model reporting and appraisal frameworks, which emphasize complete model specification, appropriate validation, and evaluation of predictive performance rather than isolated association [16,17]. The prespecified eligibility criteria therefore excluded association-only analyses without a reproducible prediction tool and local applications of established scores without substantive validation, comparison, updating, recalibration, or methodological advancement. This prediction-model-focused eligibility boundary narrowed the evidence set while providing a more methodologically coherent map of tools intended for individual-level preoperative prediction.

### 4.2. Relationship with Previous Evidence

The present findings extend the systematic review by Vannucci et al., which identified 22 model-development or validation studies through June 2020 [3]. The subsequent literature is broader not only because of additional publication years but also because the methodological spectrum has expanded to include more regression-based and regression-derived nomograms, quantitative imaging, machine-learning approaches, broader validation strategies, and safety-oriented prediction targets such as failure to achieve the CVS and the need for bailout surgery [7,8,9,10,13,14,15,53].

Despite this expansion, several methodological concerns identified in the earlier review remain evident. Definitions of difficult LC continue to vary substantially, transportability remains limited for many newly developed models, reporting is often incomplete, and evidence that prediction tools improve clinical decision-making or patient outcomes remains scarce [3,6].

### 4.3. The Prediction Target Remains a Central Source of Heterogeneity

“Difficult laparoscopic cholecystectomy” is a multidimensional construct. Adhesions, inflammatory distortion, impaired exposure, bleeding, prolonged operative duration, failure to achieve the CVS, conversion to open surgery, and subtotal cholecystectomy may coexist but do not represent equivalent clinical events [1,2,3,5,7]. A model developed to predict prolonged operative time should therefore not automatically be interpreted as a model of safety-critical anatomy or bailout risk. Conversion is particularly context-dependent, being influenced by surgeon experience, institutional practice, available resources, and thresholds for abandoning laparoscopic dissection [1,2,3]. Importantly, conversion may reflect technical difficulty but may also represent an appropriate safety decision when continued laparoscopic dissection is considered hazardous [1,2].

Future research would benefit from a core outcome framework that distinguishes direct operative-difficulty grades from prolonged operative duration, failure to achieve the CVS, conversion to open surgery, subtotal cholecystectomy, and other bailout procedures. Such standardization could improve comparability across prediction studies without collapsing clinically distinct outcomes into a single composite endpoint [3,5,7,13].

### 4.4. Validation Requires Precise Terminology and Complete Reporting

The term “validation” should specify the relationship between development and evaluation data. Apparent performance, internal resampling validation, internal split-sample validation, temporal validation, geographic external validation, and independent validation of an existing published model or score address different methodological questions [16,17]. Random division of a single source population constitutes internal split-sample validation, even when described by study authors as external validation. Prospective recruitment likewise characterizes study design and does not, by itself, establish independence from model development. Implementation and impact evaluation should therefore be reported separately from predictive-performance validation [16,17].

Discrimination, calibration, clinical utility, and clinical impact address distinct aspects of model performance. Discrimination, commonly measured by AUC, describes how well a model distinguishes patients with and without the target outcome. Calibration assesses agreement between predicted probabilities and observed outcomes. Clinical utility concerns whether using model predictions offers net benefit over alternative strategies at clinically relevant decision thresholds. Clinical impact concerns demonstrated changes in clinical management, resource use, or patient outcomes following model implementation [16,17]. A model may therefore achieve a high AUC while providing poorly calibrated risk estimates or offering little benefit for clinical decision-making.

The limited reporting of calibration and decision-curve analysis in this review restricts conclusions about the clinical readiness of available tools. Decision-curve analysis estimates potential clinical utility but does not demonstrate that implementation improves care. Similarly, explainability methods may support transparency regarding model behavior without establishing calibration or clinical benefit. These assessments complement, rather than replace, independent validation and prospective clinical-impact evaluation. Complete model specification, including equations or point systems, predictor definitions, preprocessing procedures, and accessible calculators or code where relevant, is also necessary for reproducibility and independent evaluation [16,17].

### 4.5. Machine Learning and Quantitative Imaging

Machine learning and quantitative imaging represent genuine methodological diversification, although they remain numerically uncommon compared with point-based prediction scores. Analytical methods, predictor modality, and model presentation should be distinguished conceptually. Radiomics, CT-derived variables, and ultrasonographic measurements describe predictor sources; regression and machine-learning methods describe analytical approaches used to generate predictions; and point scores or nomograms describe ways in which a prediction model may be operationalized or presented for clinical use. Potential advantages of machine learning include flexible modeling of nonlinear relationships and interactions, automated or regularized feature selection in selected algorithms, and post hoc model-interpretation methods. Recent studies have also incorporated evaluation elements that were less consistently reported in earlier score-based models, including calibration, decision-curve analysis, cross-validation, SHAP-based interpretation, and online implementation [10,13,14].

However, only four of the 59 included publications (6.8%) were categorized as machine-learning models, and their populations, prediction targets, predictor sets, and validation designs differed substantially. AUC values from these studies cannot therefore be directly compared with those reported for conventional point-based or regression-based models. The available evidence does not establish the superiority of machine-learning approaches. Their inclusion in this review reflects an emerging methodological direction rather than demonstrated comparative effectiveness or greater clinical usefulness. Machine-learning and imaging-derived models may be vulnerable to overfitting, data leakage, variability in image acquisition and segmentation, dataset shift, and inadequately characterized missing data [16,17]. New algorithms should ideally be compared with strong, interpretable conventional baselines in the same patients, for the same prediction target, and using the same evaluation dataset. Explainability methods may improve transparency regarding model behavior but cannot compensate for biased source data, poor model development, or inadequate validation. TRIPOD+AI and PROBAST+AI provide relevant contemporary frameworks for transparent reporting and methodological appraisal of such models [16,17]. These frameworks were used as contemporary interpretive reference points only. PROBAST+AI was not formally applied to the included publications, and no domain-level or overall risk-of-bias or applicability judgments were assigned.

Reporting of machine-learning development procedures was also incomplete. Although several publications described feature selection and cross-validation, classifier-specific hyperparameter tuning and safeguards against data leakage were insufficiently documented. In particular, it was not consistently clear whether preprocessing, feature selection, and class balancing were restricted to training data and repeated within resampling folds. These reporting gaps limit reproducibility and confidence in performance estimates without establishing that the procedures were omitted or that leakage occurred.

### 4.6. Clinical Implications

Preoperative risk stratification has several plausible clinical applications, including allocation of technically demanding cases to appropriately experienced surgeons, planning supervision of trainees, operating-room scheduling, patient counselling, preparation of adjunctive imaging or equipment, and anticipation of bailout strategies [1,2,3,5]. However, the practical value of a prediction model depends on a clearly defined link between predicted risk and predefined clinical action. Decision thresholds should therefore reflect the relative consequences of false-negative and false-positive classifications rather than being selected solely to maximize an ROC-derived performance statistic [16,17]. For example, a validated score based on routinely collected preoperative information could help identify patients requiring senior surgical supervision, extended operating-room allocation, or advance preparation for potential bailout procedures; however, such applications require locally appropriate thresholds and prospective evaluation before routine adoption.

At present, the available evidence supports prediction tools primarily as structured aids for operative planning and research rather than as autonomous decision systems. Before routine clinical implementation, a model should have a reproducible specification, feasible and consistently defined input variables, adequate calibration in the intended population, acceptable transportability, interpretable output, and demonstrated incremental clinical value beyond routine assessment [16,17]. The limited implementation evidence identified in this review [6,65] further indicates that prospective impact studies are needed to determine whether model-guided risk stratification changes surgical planning, resource allocation, operative safety, or patient outcomes.

### 4.7. Priorities for Future Research

Future research should establish a consensus core outcome framework that distinguishes direct operative difficulty from prolonged operative duration, conversion to open surgery, failure to achieve the CVS, subtotal cholecystectomy, and other bailout outcomes [1,2,3]. Studies should use adequately sized, consecutive, and preferably multicenter cohorts with prespecified outcomes and predictor definitions; classify apparent performance, internal resampling validation, internal split-sample validation, temporal validation, geographic external validation, and independent validation of existing published models or scores precisely; and report calibration alongside discrimination [16,17].

New nomogram-based, machine-learning, and imaging-derived models should be compared directly with strong, interpretable baselines in the same patients and for the same prediction target. Complete equations, scoring rules, preprocessing pipelines, calculators, or code should be made available to permit independent reproduction and evaluation [16,17]. Model assessment should extend beyond AUC to clinically meaningful thresholds, net benefit, and prospective impact studies examining whether model-guided care changes surgeon allocation, resource use, conversion or bailout decisions, operative safety, or patient outcomes [6,16,17].

### 4.8. Strengths and Limitations

A major strength of this review is its explicit prediction-model-focused eligibility framework derived from the registered OSF protocol. Model roles were distinguished from the predominant prediction-tool category: development, substantive validation, comparison, updating, and implementation were charted separately from point-based prediction scores, regression-based probability models, regression-derived nomograms, machine-learning models, and imaging-derived quantitative prediction tools. Predictor modality was recorded as a separate dimension. Studies limited to isolated predictors, association-only analyses without a reproducible prediction tool, or non-substantive local reapplication of established scores were excluded. The review also retained study-specific definitions of difficult LC rather than imposing a retrospective review-level definition, allowing heterogeneity and evolution in prediction targets to be mapped directly. Validation terminology was standardized according to the relationship between development and evaluation data, reducing potential misclassification arising from inconsistent terminology in the source literature.

Several limitations should be acknowledged. First, the literature search was restricted to PubMed/MEDLINE, Scopus, and Web of Science Core Collection, as prespecified in the registered OSF protocol. Although these databases provide broad coverage of biomedical and multidisciplinary literature, the absence of Embase may have resulted in the omission of relevant studies indexed exclusively in that database. Additionally, the restriction to English-language publications and the absence of dedicated grey-literature searching may have limited the comprehensiveness of the evidence map. These limitations should be considered when interpreting the findings. Backward citation searching, although initially planned, was removed through a documented protocol amendment. In addition, because the search strategy was centered on terminology related to difficult laparoscopic cholecystectomy and conversion rather than exhaustively enumerating every possible safety-oriented manifestation of operative difficulty, studies using exclusively alternative terminology such as bailout procedures or failure to achieve the critical view of safety may have been missed. A preliminary post hoc PubMed exploration using additional safety-related terminology suggested that potentially relevant publications may have been missed. Because this exploration was not completed as a formal sensitivity search with independent eligibility assessment, its results were not incorporated into the final evidence synthesis. Consequently, the comprehensiveness of the evidence map remains limited by the prespecified search strategy. Second, four potentially relevant reports could not be retrieved in full text and therefore did not undergo eligibility assessment; in accordance with the prespecified protocol, study authors were not contacted. Third, substantial heterogeneity in prediction targets, populations, predictor definitions, model structures, thresholds, and validation procedures precluded meaningful quantitative pooling or indirect cross-study ranking. Fourth, the publication/report was the unit of descriptive analysis, and some included publications may have drawn on partially overlapping patient populations. Consequently, the 59 analytic entries should not be interpreted as 59 necessarily independent patient cohorts, and such overlap may reduce the effective independence of the evidence base.

Finally, no formal study-level risk-of-bias, applicability, or certainty-of-evidence assessment was performed, consistent with the descriptive objectives of this scoping review. Although contemporary prediction-model frameworks such as TRIPOD+AI and PROBAST+AI informed the interpretation of reporting and methodological features, they were not applied as formal appraisal instruments. Observations regarding calibration, missing-data handling, model specification, validation design, transportability, and clinical readiness should therefore be interpreted as descriptive methodological mapping rather than formal judgments of study quality or risk of bias. Reporting missing-data handling and complete model specification was also inconsistent, but these features were not captured as binary study-level summary variables and were therefore not quantified retrospectively.

## 5. Conclusions

Across 59 included studies, preoperative prediction of difficult LC evolved from predominantly point-based clinical and imaging scores toward greater diversity in analytical methods, predictor modalities, and model presentation, including regression-based models, regression-derived nomograms, machine-learning approaches, and quantitative imaging tools. Point-based prediction scores nevertheless remained dominant (45/59, 76.3%), and recent methodological diversification has not eliminated longstanding concerns about heterogeneous prediction targets and limited transportability.

The principal challenge is therefore no longer the absence of proposed tools, but determining which models provide reproducible, calibrated, transportable, and clinically actionable information. Standardized outcomes, transparent model specification, temporal validation and geographic external validation, independent validation of existing published models and scores, direct comparison with interpretable baselines, and prospective impact evaluation should take priority over continued proliferation of locally derived scores. Until these requirements are met, most models are best regarded as candidate tools for structured risk stratification and surgical planning rather than established clinical decision-support tools.

## Figures and Tables

**Table 1:** Distribution of the 59 included publications by publication period.

Publication Period	Included Publications, *n*	% of 59	Publications/Year
2010–2014	3	5.1	0.6
2015–2019	9	15.3	1.8
2020–2024	25	42.4	5.0
2025–31 July 2026	22	37.3	13.9

Annualized publication counts were calculated by dividing the number of publications by the duration of each observation window: five years for 2010–2014, 2015–2019, and 2020–2024, and 19/12 years for January 2025–July 2026. These values describe publication rates within the observed periods and are not projections. Percentages may not sum to exactly 100% because of rounding.

**Table 2:** Predominant prediction-tool category among the 59 included publications. Each study was assigned one mutually exclusive category according to its principal predictive method; predictor modality and model role were charted separately.

Predominant Prediction-Tool Category	Included Publications, *n*	% of 59
Point-based prediction score	45	76.3
Regression-based prediction model	4	6.8
Regression-derived nomogram	5	8.5
Machine-learning model	4	6.8
Imaging-derived quantitative prediction tool	1	1.7

Percentages may not sum to exactly 100% because of rounding.

**Table 3:** Methodological maturity, predictor modalities, and prediction-target features across the 59 included publications.

Characteristic	Included Publications, *n*	% of 59
Validation features in model-development publications *		
Internal holdout evaluation	7	11.9
Resampling as the principal internal validation, without a separate holdout or external cohort	3	5.1
New tool evaluated in a cohort with a geographic external component	4	6.8
Mixed temporal/geographic validation cohort (included in the preceding row)	1	1.7
Evaluated clinical implementation *		
Prospective evaluation during clinical use of a score	2	3.4
Controlled clinical-impact study	0	0.0
Methodological reporting		
Calibration assessment reported for a prediction model	9	15.3
Decision-curve analysis reported	7	11.9
Formal explainability analysis reported (SHAP)	2	3.4
Predictor modalities (non-mutually exclusive) *		
Clinical/history variables	50	84.7
Laboratory/inflammatory markers	19	32.2
Conventional ultrasonography/sonography	44	74.6
CT/cross-sectional imaging	5	8.5
Advanced ultrasonography	1	1.7
Radiomics	1	1.7
Conventional imaging, modality not further specified	4	6.8
Multimodal predictor combination	51	86.4
Prediction-target features (non-mutually exclusive) *		
Generic/ordinal difficult-LC or surgical-difficulty construct without a more specific target label	26	44.1
Conversion to open surgery	13	22.0
Operative-time-based difficulty	5	8.5
Objective intraoperative grading (Nassar/Parkland or equivalent objective grade)	6	10.2
CVS-related difficulty/failure to achieve CVS	2	3.4
Bailout procedure	2	3.4
Composite difficult-LC/technical-difficulty endpoint	6	10.2
Adhesion-staging endpoint	1	1.7
Adversity-related operative endpoint	1	1.7

* Validation rows describe features of model-development publications and are not an exhaustive or mutually exclusive partition of the evidence set. The mixed temporal/geographic cohort is included among the four publications with a geographic external validation component. Some publications also used resampling during model development. Independent evaluation of existing scores and apparent performance of new or modified tools are detailed separately in Supplementary Table S1. Implementation denotes prospectively evaluated use in a management pathway or routine theatre booking; it does not establish a controlled clinical benefit. Predictor modalities and prediction-target features are not mutually exclusive. All percentages use 59 publications as the denominator. Calibration reporting refers to the developed prediction model and excludes goodness-of-fit tests for secondary association models. * CT, computed tomography; CVS, critical view of safety; LC, laparoscopic cholecystectomy; n, number of publications; SHAP, SHapley Additive exPlanations.

**Table 4:** Clinical applicability, validation, and implementation of selected preoperative prediction tools for difficult laparoscopic cholecystectomy.

Study/Prediction Target	Required Preoperative Information/Tool Format	Validation Evidence	Clinical Applicability and Limitations
Nassar et al., 2020 [5]—Objective operative difficulty	Clinical history, ASA class, biliary diagnosis, routine US, ERCP history; 0–19-point score	Internal and geographic external validation; external AUC 0.789	Reproducible point score using routinely available information; potential for risk stratification and surgeon allocation; clinical impact not evaluated
Hu et al., 2018 [28]—Conversion to open surgery	Clinical history, BMI and US findings; five-variable logistic prediction model	Independent geographic external validation; AUC 0.870	Calculable from routine preoperative data; no additional advanced imaging required; clinical impact not established
Kapoor et al., 2023 [8]—Operative adhesions	Advanced US elastography (ARFI/VTI); quantitative adhesion-detection system	Same-cohort evaluation only; inconsistent reported AUC estimates	Requires specialized ultrasound assessment; feasibility and transportability remain uncertain
Sun et al., 2025 [10]—Difficult LC	Clinical, laboratory and US information combined with CT radiomics; random-forest model and online calculator	Internal split-sample validation; AUC 0.874	Requires radiomics extraction and computational processing; no geographic external validation or demonstrated clinical impact
Huang et al., 2025 [53]—Surgical difficulty	Clinical factors, inflammatory indices and preoperative US, including Calot-triangle assessment; nomogram	Internal split-sample validation; AUC 0.863	Graphical prediction tool; reproducibility of specialized US assessments requires clarification
Suzuki et al., 2025 [7]—Bailout surgery	Clinical information, TG18 severity, CRP, biliary intervention history and imaging; nomogram	Internal split-sample validation; AUC 0.769	Relevant to anticipating bailout requirements; no geographic external validation or evaluated model-guided pathway
Wu et al., 2025 [13]—Conversion in acute cholecystitis	CRP, ASA class, TG18 severity, stone location and surgeon experience; nomogram	Geographic external validation; AUC 0.833	May inform risk-adapted operative planning; requires further confirmation of coding and clinical decision thresholds
Yang et al., 2026 [14]—Difficult LC after PTGBD	Clinical history, laboratory values, US findings and drainage-to-surgery intervals; machine-learning calculator	Geographic external validation; AUC 0.868	Online calculator available; applicability restricted to patients undergoing LC after PTGBD; clinical impact not evaluated
Zhang et al., 2025 [6]—Operative difficulty and risk-adapted management	Clinical and US variables, combined with surgeon experience; point score and management pathway	Development AUC 0.893; prospective implementation cohort	Score-guided surgical allocation and operative pathways evaluated; absence of a concurrent usual-care control limits causal conclusions
Ayantunde et al., 2026 [65]—Operative difficulty and theatre planning	Routine clinical, laboratory and imaging information; Traffic Light preoperative score	New-site prospective evaluation; AUC 0.797	Used for theatre scheduling and surgeon review; workflow implementation documented, but controlled clinical benefit not demonstrated

ARFI, acoustic radiation force impulse; ASA, American Society of Anesthesiologists; AUC, area under the receiver operating characteristic curve; BMI, body mass index; CRP, C-reactive protein; CT, computed tomography; ERCP, endoscopic retrograde cholangiopancreatography; LC, laparoscopic cholecystectomy; PTGBD, percutaneous transhepatic gallbladder drainage; TG18, Tokyo Guidelines 2018; US, ultrasonography; VTI, virtual touch imaging. These publications were selected to illustrate differences in prediction targets, data requirements, validation maturity, and clinical application. Practical feasibility is inferred descriptively from the reported inputs and model format; it was not formally tested or ranked. AUC values refer to different outcomes, populations, and validation designs and should not be compared indirectly. Documented implementation does not establish a causal clinical benefit.

## Data Availability

The data supporting the findings of this review are available within the article and its Supplementary Materials. Additional study-level charting data and the registered review protocol are available on the Open Science Framework (OSF doi: 10.17605/OSF.IO/GQYH8).

## Supplementary Materials

The following supporting information can be downloaded at https://www.mdpi.com/article/10.3390/medicina62102042/s1: Table S1: Study-level characteristics, prediction-tool categories, predictor modalities, validation classifications, and methodological features of the 59 included publications; Table S2: Full-text retrieval and eligibility audit [67,68,69,70,71]; Supplementary File S1: Completed PRISMA-ScR checklist.

## Abbreviations

The following abbreviations are used in this manuscript:

ADS	Adhesion detection system
AI	Artificial intelligence
ARFI	Acoustic radiation force impulse
AUC	Area under the receiver operating characteristic curve
CT	Computed tomography
CVS	Critical view of safety
JBI	Joanna Briggs Institute
LC	Laparoscopic cholecystectomy
ML	Machine learning
OSF	Open Science Framework
PRISMA-ScR	Preferred Reporting Items for Systematic Reviews and Meta-Analyses Extension for Scoping Reviews
PROBAST+AI	Prediction model Risk Of Bias Assessment Tool + Artificial Intelligence
PTGBD	Percutaneous transhepatic gallbladder drainage
ROC	Receiver operating characteristic
SHAP	SHapley Additive exPlanations
TRIPOD+AI	Transparent Reporting of a multivariable prediction model for Individual Prognosis Or Diagnosis + Artificial Intelligence
VTI	Virtual touch imaging
