# Supplementary material for: Preoperative Prediction of Difficult Laparoscopic Cholecystectomy: Evolution, Validation, and Emerging Machine-Learning Approaches—A Scoping Review
Source: Medicina (Kaunas). 2026 Oct 21;62(10):2042. doi: 10.3390/medicina62102042 (PMC13707204; doi:10.3390/medicina62102042)
Supplement: Supplementary file 1 [file medicina-62-02042-s001.zip › Completed PRISMA-ScR checklist.pdf]

Preferred Reporting Items for Systematic reviews and Meta-Analyses extension for Scoping Reviews (PRISMA-ScR) Checklist

**TITLE**			
**Title**	**1**	Identify the report as a scoping review.	p. 1
**ABSTRACT**			
**Structured summary**	**2**	Summarize the rationale, sources, selection, charting, findings and conclusions.	p. 2
**INTRODUCTION**			
**Rationale**	**3**	Explain the knowledge gap and why scoping review methods fit.	pp. 2–3 (Introduction)
**Objectives**	**4**	State review questions and relevant population–concept–context elements.	pp. 3–4 (Introduction)
**METHODS**			
**Protocol and registration**	**5**	Give protocol access details and any registration identifier.	p. 5 (2.1; OSF DOI)
**Eligibility criteria**	**6**	Specify eligible sources, publication years, language and study restrictions.	pp. 5–6 (2.2–2.3); p. 6 (2.4)
**Information sources**	**7**	Identify searched resources and the last search date.	pp. 6–7 (2.4)
**Search**	**8**	Provide reproducible database queries with restrictions.	pp. 6–7 (2.4)
**Selection of sources of evidence**	**9**	Describe title/abstract and full-text screening processes.	p. 7 (2.5)
**Data charting process**	**10**	Explain extraction, reviewer independence and discrepancy resolution.	p. 8 (2.6)
**Data items**	**11**	Identify extracted variables and classification assumptions.	pp. 8–9 (2.6–2.7); Table S1
**Critical appraisal of individual sources**	**12**	If performed, state appraisal method and use in synthesis.	N/A — not performed; p. 9 (2.7)
**Synthesis of results**	**13**	Describe approaches used to organize and summarize charted data.	p. 9 (2.7)
**RESULTS**			
**Selection of sources of evidence**	**14**	Report screened, retrieved, excluded and included records.	pp. 9–10 (3.1; Figure 1; Table S2)
**Characteristics of sources of evidence**	**15**	Present charted characteristics and citations for all included sources.	pp. 12–17 (3.4–3.7); Table S1, Panels A–C
**Critical appraisal within sources of evidence**	**16**	If performed, report appraisal findings.	N/A — not performed; pp. 9, 22
**Results of individual sources of evidence**	**17**	Report relevant results for individual included sources.	Table S1, Panels A–D; pp. 16–17 (Table 4)
**Synthesis of results**	**18**	Synthesize charted evidence in relation to the review objectives.	pp. 10–17 (3.2–3.8; Tables 1–4, Figure 2)
**DISCUSSION**			

**Summary of evidence**	**19**	Interpret major evidence patterns and implications for clinical readers.	pp. 18–21 (4.1–4.7)
**Limitations**	**20**	Discuss limitations of the review process.	pp. 21–23 (4.8)
**Conclusions**	**21**	Link conclusions to objectives and future research needs.	p. 23 (5)
**FUNDING**			
**Funding**	**21**	Report review and included-source funding, and funder roles.	p. 21 no external funding

JBI = Joanna Briggs Institute; PRISMA-ScR = Preferred Reporting Items for Systematic reviews and Meta-Analyses extension for Scoping Reviews.

Reference: Tricco AC, Lillie E, Zarin W, O'Brien KK, Colquhoun H, Levac D, et al. PRISMA Extension for Scoping Reviews (PRISMAScR): Checklist and Explanation. Ann Intern Med. 2018;169:467–473. doi: 10.7326/M18-0850.
