# Supplementary material for: Preoperative Prediction of Difficult Laparoscopic Cholecystectomy: Evolution, Validation, and Emerging Machine-Learning Approaches—A Scoping Review
Source: Medicina (Kaunas). 2026 Oct 21;62(10):2042. doi: 10.3390/medicina62102042 (PMC13707204; doi:10.3390/medicina62102042)
Supplement: Supplementary file 1 [file medicina-62-02042-s001.zip › Supplementary_Table_S1.pdf]

Supplementary Table S1. Study-level characteristics, prediction-tool categories, predictor modalities, validation classifications, and methodological features of the 59 included publications.

The evidence map is presented in four linked panels. Study numbers and manuscript reference numbers are consistent across panels. Panel A describes populations, study design, and confirmed or possible cohort overlap; Panel B describes predictors, model roles, and validation; Panel C describes performance, reporting, and clinical translation; and Panel D summarizes machine-learning development procedures and reporting for the four publications categorized as machine-learning models.

Missing information. NR = not reported in the examined article. A separate supplement cited but unavailable for assessment is identified explicitly in the relevant entry. Source discrepancies are noted without replacing reported estimates with reconstructed values.

Model type represents the predominant tool category. Predictor modalities are recorded separately. Validation describes the relationship between development and evaluation data; prospective recruitment and decision-curve analysis do not establish external validation or clinical impact. Performance estimates should not be ranked indirectly across different populations, outcomes, thresholds, or validation designs.

Cohort overlap. The unit of analysis is the publication/report; 59 publications do not necessarily represent 59 independent patient cohorts. Overlap is assessed between included publications using the reported cohort/database, recruiting institutions, recruitment dates, and eligibility criteria. Confirmed overlap denotes explicit reuse of the same underlying cohort/database, without implying identical analytic samples. Possible overlap denotes compatible recruitment at the same institution without confirmation of shared patients. No overlap identified based on available information does not establish cohort independence, particularly where recruiting sites or dates are incompletely reported. Reuse or validation of the same prediction score alone does not imply patient overlap. The shared patient count is not estimated when unreported.

Panel A Study characteristics and outcome definitions

Study and reference	Country	Study setting	Sample size n	Clinical population	Study design	Elective or emergency status	Prediction target and outcome definition	Underlying cohort / possible cohort overlap
01 Kareem et al. 2010 [21]	Iraq	Rizgary Teaching and Hawler Private Hospitals, Erbil; single surgeon	200	Symptomatic gallstones; observed age 16–79	Prospective observational; October 2005–March 2007	NR; ultrasound one day before surgery	Difficult nonconverted LC: ≥30 min from port insertion to cystic duct/artery clipping; conversion analyzed separately.	No overlap identified based on available information.
02 Vyas et al. 2013 [20]	India	Vardhman Mahavir Medical College/Safdarjang Hospital, New Delhi; single unit	100	Gallstone disease; eligibility >12, observed age 20–63; acute cholecystitis excluded	Prospective comparative score study; October 2011–April 2013	NR; scheduled LC after preoperative assessment	Any difficulty in access/dissection/adhesiolysis/extraction or operative time >90 min; open conversion also recorded.	No overlap identified based on available information.
03 Gupta et al. 2013 [22]	India	Lady Hardinge Medical College/Dr R.M.L. Hospital, Delhi; single surgical unit	210	Symptomatic gallstone disease; current acute-cholecystitis admission excluded	Nonrandomized prospective validation; November 2010–October 2012	NR; current acute attacks excluded	Easy <60 min without spillage/injury; difficult 60–120 min and/or spillage/injury without conversion; very difficult >120 min or conversion.	No overlap identified based on available information.
04 Joshi et al. 2015 [23]	Nepal	Kathmandu Medical College Teaching Hospital; one surgical unit	100	Symptomatic cholelithiasis; observed age 14–78	Prospective observational cross-sectional development; August–December 2014	Elective	Easy ≤45 min; difficult 46–90 min, spillage/duct injury without conversion; very difficult >120 min or conversion (91–120 min gap).	No overlap identified based on available information.

Study and reference	Country	Study setting	Sample size n	Clinical population	Study design	Elective or emergency status	Prediction target and outcome definition	Underlying cohort / possible cohort overlap
05 Bourgouin et al. 2016 [4]	France	Sainte-Anne Military Teaching Hospital, Toulon; tertiary urban center	420 analyzed (644 initially identified)	Adults with biliary colic, acute or previous cholecystitis; CBD stones excluded	Retrospective development; May 2010–May 2015	Elective and acute/emergency presentations	Operative time ≥1.5× each surgeon's median uncomplicated elective time, or open conversion; 148 difficult.	No overlap identified based on available information.
06 Sutcliffe et al. 2016 [24]	United Kingdom	CholeS prospective cohort across 166 UK hospitals	8,820 (6,615 development / 2,205 validation)	Patients undergoing LC for symptomatic biliary disease	Prospective multicenter cohort analysis; March–April 2014	Elective, delayed and acute	Conversion from laparoscopic to open; 297 events (3.4%).	**Confirmed overlap** CholeS cohort (March-April 2014), also analyzed by Nassar et al. 2020 [5] (study 15). Different analytic samples/targets; exact shared patient count NR.
07 Beksac et al. 2016 [25]	Turkey	Hacettepe University General Surgery, Ankara; single center	208 case-control (104 converted + 104 controls), from 1,335 LC starts	Benign gallbladder pathology; sampled nonconversions matched in number only	Retrospective case-control development; August 2006–January 2011	NR; includes daytime/nighttime surgery and interval post-treatment cases	Conversion to open surgery.	No overlap identified based on available information.
08 Siddiqui et al. 2017 [26]	India	Single experienced surgeon; Aligarh affiliations. Recruiting institution not explicitly named.	300	Symptomatic gallstone disease; age 19–75	Retrospective score development; January 2008–March 2011	Elective; emergency cases excluded	Any operative time >60 min, biliary leakage, duct/arterial injury or conversion.	No overlap identified based on available information.
09 Saleem & Abdallah 2018 [27]	Egypt	Aswan University Hospital, general surgery; one center	100	Symptomatic cholelithiasis; age 19–70; acute cholecystitis excluded	Prospective validation; January 2015–December 2017	Elective	Easy <60 min without spillage/injury; difficult 60–120 min and/or spillage/injury without conversion; very difficult >120 min or conversion.	No overlap identified based on available information.
10 Hu et al. 2018 [28]	Australia	Townsville Hospital, Queensland; level-4 teaching hospital	1,035	All LC starts; no age restriction; retrieved cohort age 6–94	Retrospective geographic validation; January 2013–December 2016	Elective and emergency; no admission-status exclusion	Conversion to open cholecystectomy; 43 events (4.2%).	No overlap identified based on available information.
11 Veeranki & Togale 2018 [66]	India	KLE Dr Prabhakar Kore Hospital/Research Centre, Belagavi; one center	30	Acute/chronic calculous cholecystitis requiring cholecystectomy	Prospective descriptive cross-sectional validation; January–December 2017	NR; scored one day before surgery	Easy <60 min without spillage/injury; difficult 60–120 min and/or spillage/injury without conversion; very difficult >120 min or conversion.	No overlap identified based on available information.

Study and reference	Country	Study setting	Sample size n	Clinical population	Study design	Elective or emergency status	Prediction target and outcome definition	Underlying cohort / possible cohort overlap
12 Wennmacker et al. 2019 [29]	Australia	Royal North Shore Hospital, Sydney; tertiary upper-GI unit	185	Adults with acute biliary presentation through ED; 59% acute cholecystitis	Retrospective analysis of prospectively collected data; July 2015–February 2018	Emergency during same admission or semi-elective within one week of discharge	Modified intraoperative difficulty grades III–IV versus I–II; 113 complicated cases.	No overlap identified based on available information.
13 AbdelDayem et al. 2020 [30]	United Kingdom	Prince Charles Hospital, Merthyr Tydfil; district general hospital	280	Gallstone disease; observed age 10–88	Retrospective development; June 2013–June 2014	Elective	Operative time ≥63 min and/or conversion; 11 conversions (3.9%).	No overlap identified based on available information.
14 Boraii & Abdelaziz 2020 [31]	Egypt	National Hepatology and Tropical Medicine Research Institute, Cairo; tertiary center	60	Symptomatic cholelithiasis; age 20–74	Prospective observational validation; November 2018–June 2019	Elective; emergency cases excluded	Easy <60 min; difficult 60–120 min; very difficult >120 min or conversion. Spillage alone did not define difficulty.	No overlap identified based on available information.
15 Nassar et al. 2020 [5]	United Kingdom	CholeS: 166 hospitals; reference series: one surgeon across four hospitals	8,755 + 4,089; score available in 6,188 development / 2,064 internal / 3,340 external	Patients undergoing LC for benign biliary disease	Analysis of prospectively maintained cohorts; CholeS March–April 2014; reference February 1992–July 2014	Elective, delayed and emergency in both cohorts	Nassar operative grades III–V difficult; CholeS used I–IV, reference I–V with IV/V combined.	**Confirmed overlap** CholeS component shared with Sutcliffe et al. 2016 [24] (study 06); additional single-surgeon reference cohort (1992-2014). Exact shared patient count NR.
16 Kumar et al. 2021 [32]	India	SS Hospital, Banaras Hindu University, Varanasi; one center	209	Symptomatic gallstone disease; observed age 14–74	Prospective observational; September 2016–July 2018	Elective	Easy <60 min/no major findings; difficult 60–120 min, adhesions/impaction/spillage or duct injury; very difficult >120 min or conversion.	No overlap identified based on available information.
17 Goyal et al. 2021 [33]	India	Maharishi Markandeshwer Medical College/Hospital, Solan; single surgeon	102	Symptomatic cholelithiasis	Prospective comparative score study; July–December 2019	NR; admitted day before surgery	Any operative time >60 min, adhesions or bile/stone spillage; conversion recorded separately.	No overlap identified based on available information.
18 Wibowo et al. 2022 [34]	Indonesia	Ulin Referral Hospital, Banjarmasin; one center	134	Gallstone LC; eligibility/urgency details NR	Nonrandomized retrospective descriptive/cross-sectional; January 2015–December 2020	NR	Operative duration only: <60, 60–120, >120 min.	No overlap identified based on available information.

Study and reference	Country	Study setting	Sample size n	Clinical population	Study design	Elective or emergency status	Prediction target and outcome definition	Underlying cohort / possible cohort overlap
19 Matsumoto et al. 2022 [35]	Japan	Jikei University Daisan Hospital (development); Daisan + Jikei University Hospital (validation)	70 + 65	Acute cholecystitis after PTGBD; intended LC	Retrospective; development 2014–2019; validation 2020–2021 locally and 2015–2020 at second hospital	LC after PTGBD; urgency NR	Bailout: open conversion or laparoscopic subtotal cholecystectomy (fenestrating/reconstituting).	No overlap identified based on available information.
20 Ramírez-Giraldo et al. 2022 [36]	Colombia	Hospital Universitario Mayor–Méderi, Bogotá; one center	319	Adults with benign biliary disease	Retrospective diagnostic validation; February–June 2020	Elective/delayed/emergency admission types evaluated	Nassar III–IV difficult versus I–II; operative record and photographic documentation.	**Possible overlap** Same hospital as Ramírez-Giraldo et al. 2023 [45] (study 30). February-June 2020 falls within January 2014-December 2021; patient-level overlap unconfirmed.
21 Reddy et al. 2022 [37]	India	RL Jalappa Hospital, Kolar; one surgeon	66 analyzed (70 abstract)	Gallstone disease; precise adult/urgency eligibility NR	Prospective comparative; November 2018–October 2020	NR for study cohort	Easy <60 min without spillage/injury; difficult 60–120 min and/or spillage/injury without conversion; very difficult >120 min or conversion.	No overlap identified based on available information.
22 Paul et al. 2022 [38]	India	St John's Medical College Hospital, Bengaluru; one center	132	Acute calculous cholecystitis; pediatric/pregnant /CBD-stone/cholangitis cases excluded	Prospective observational; December 2018–December 2020	Early LC within 7 days of symptoms	Time <60 min easy; 60–120 min or spillage/duct injury difficult; >120 min or conversion very difficult.	No overlap identified based on available information.
23 Chen et al. 2022 [39]	China	Single hospital cohort, Hefei; precise recruiting institution not specified	201	Adults with gallstones/polyps or acalculous cholecystitis	Retrospective cohort; January 2018–November 2019	NR	Operative time ≥75th percentile (70 min); 53 difficult cases.	No overlap identified based on available information.
24 Kotb et al. 2023 [40]	Egypt	Assiut University Hospitals; one experienced operator	100	Adults with symptomatic gallstones	Prospective nonrandomized observational; January–December 2019	Elective	Operative time longer than the surgeon's mean; reported mean 82.50 min, 40 difficult cases.	No overlap identified based on available information.
25 Mazni et al. 2022 [41]	Indonesia	Dr Cipto Mangunkusumo Central General Hospital, Jakarta; single center	163	Predominantly symptomatic gallstones (88.3%); 11.7% cholecystitis	Retrospective validation; January 2018–December 2019	NR	Conversion to open cholecystectomy; 5 events (3.1%).	No overlap identified based on available information.

Study and reference	Country	Study setting	Sample size n	Clinical population	Study design	Elective or emergency status	Prediction target and outcome definition	Underlying cohort / possible cohort overlap
26 Morsy et al. 2023 [42]	Egypt	Assiut University Hospitals; general surgery department	50	Adults with uncomplicated chronic or acute-on-chronic calculous cholecystitis	Observational cohort with preoperative collection; April–October 2022	Elective	Easy <60 min without spillage/injury; difficult 60–120 min and/or spillage/injury without conversion; very difficult >120 min or conversion.	No overlap identified based on available information.
27 Kapoor et al. 2023 [8]	India	Advanced Diagnostics and two surgical hospitals, Amritsar	50	Consecutive cholelithiasis; recent acute cholecystitis/pancreatitis excluded	Prospective paired diagnostic comparison (title: case-control); August 2021–January 2022	Planned LC; urgency NR	Operative adhesion classes II–III versus I (Zühlke-based), incorporating dissection difficulty and operative time.	No overlap identified based on available information.
28 Trehan et al. 2023 [43]	India	Dayanand Medical College and Hospital, Ludhiana; one center	120	Acute/chronic cholecystitis or symptomatic cholelithiasis	Prospective observational; January 2020–June 2021	Elective/interval LC (authors)	Easy <60 min without spillage/injury; difficult 60–120 min and/or spillage/injury without conversion; very difficult >120 min or conversion.	No overlap identified based on available information.
29 Lattoo et al. 2023 [44]	India	District Hospital Anantnag, Kashmir; rural center	327	Scheduled gallstone LC; age ≥15; active acute cholecystitis/CBD stones excluded	Prospective analytical; September 2016–September 2018	Scheduled LC; urgency NR	Easy <60 min without spillage/injury; difficult 60–120 min and/or spillage/injury without conversion; very difficult >120 min or conversion.	No overlap identified based on available information.
30 Ramírez-Giraldo et al. 2023 [45]	Colombia	Hospital Universitario Mayor–Méderi, Bogotá; 35 surgeons	635	Adults with benign biliary disease	Retrospective diagnostic comparison; January 2014–December 2021	Elective/delayed/emergency cases	Spanish consensus: injury, poor anatomical visualization, severe Calot inflammation, conversion, Mirizzi, scleroatrophic GB or pericholecystic abscess.	**Possible overlap** Same hospital as Ramírez-Giraldo et al. 2022 [36] (study 20); recruitment periods overlap. Shared patients/extent of overlap unconfirmed.
31 Mishima et al. 2024 [46]	Japan	Ageo Central General Hospital, Saitama; one center	201 (48 non-CVS)	Consecutive acute cholecystitis cases	Observational cohort analysis; January 2019–December 2021; collection timing NR	Early LC within 7 days of onset	Failure to achieve CVS by intraoperative consensus; bailout follows unsafe dissection.	No overlap identified based on available information.
32 Serrano-González et al. 2024 [47]	Venezuela	Dr Luis Razetti University Hospital, Barcelona; one center	40	Adults 18–80 with gallstone disease	Prospective cohort; September–December 2023	29 elective; 11 emergency	Parkland operative grading; difficult grades III–V versus I–II.	No overlap identified based on available information.

Study and reference	Country	Study setting	Sample size n	Clinical population	Study design	Elective or emergency status	Prediction target and outcome definition	Underlying cohort / possible cohort overlap
33 Stoica et al. 2024 [48]	Romania	Emergency University Hospital Bucharest, fourth surgical department; one team	255	Adults with acute calculous cholecystitis	Retrospective cohort; June 2019–June 2023	Emergency admission; LC within 96 h during same admission	Spanish consensus DLC: poor anatomical visualization, severe Calot inflammation, bile duct injury, conversion, Mirizzi, scleroatrophic GB or pericholecystic abscess.	No overlap identified based on available information.
34 Acharya et al. 2024 [49]	Nepal	Chitwan Medical College and Teaching Hospital, Bharatpur	116	Adults with cholelithiasis; CBD stones/jaundice/abnormal LFTs excluded	Prospective observational; July 2023–March 2024	Elective	Any: time >60 min, bile/stone spillage, duct/artery injury or open conversion.	No overlap identified based on available information.
35 Wei et al. 2024 [9]	China	Shanghai Tenth People's Hospital; one center	127 (36 difficult)	Acute cholecystitis after PTGBD	Retrospective cohort; January 2016–July 2022	Elective LC after PTGBD/stabilization (Discussion)	Any: operative duration >90 min, blood loss >100 mL or conversion.	No overlap identified based on available information.
36 Martínez-Mier et al. 2024 [50]	Mexico	IMSS Specialty Hospital No.14, Veracruz; single center	367	Adults with benign biliary disease	Prospective cohort; January 2020–December 2023	291 elective; 12 delayed; 64 emergency	Primary: Nassar III–IV. Secondary outcome-based difficulty: poor anatomy/severe inflammation, time >120 min, conversion, injury or reintervention.	No overlap identified based on available information.
37 Khan et al. 2024 [51]	Pakistan	Hayatabad Medical Complex, Peshawar; tertiary center	68	Adults with symptomatic cholelithiasis; pancreatitis allowed	Prospective enrollment; cross-sectional study; June–December 2022	NR	Conversion, time >60 min, vascular/biliary injury, significant bleeding or synthetic hemostat use; modified Nassar assessment also described.	No overlap identified based on available information.
38 Sun et al. 2025 [10]	China	Affiliated Hospital of Qingdao University; one center	2,055 (1,439 / 616)	Acute/chronic cholecystitis; preoperative CT within 7 days; prior drainage excluded	Retrospective cohort; March 2018–March 2023	NR	DLC composite score ≥4: time, conversion, intraoperative complications, blood loss, drainage and hospital stay (Table 1).	No overlap identified based on available information.
39 Agrawal et al. 2025 [52]	India	Nil Ratan Sircar Medical College, Kolkata; tertiary referral center	25 (17 training / 8 test)	20 acute and 5 acute-on-chronic cholecystitis cases	Prospective pilot with model development; January 2021–February 2022	22 urgent (<48 h); 3 elective (>48 h), as defined by authors	Expert-surgeon ground truth: easy, moderate, difficult, very difficult; operative action-based assessments.	No overlap identified based on available information.

Study and reference	Country	Study setting	Sample size n	Clinical population	Study design	Elective or emergency status	Prediction target and outcome definition	Underlying cohort / possible cohort overlap
40 Zia et al. 2025 [11]	Pakistan	Combined Military Hospital/NUMS, Rawalpindi; one center	153	Symptomatic gallbladder disease; age 18–60	Cross-sectional validation; February 2022–March 2023	NR	Any: operative time >60 min, bile/stone spillage, duct/artery injury or open conversion.	No overlap identified based on available information.
41 Huang et al. 2025 [53]	China	Mianyang Hospital of Traditional Chinese Medicine; one center	362 (253 / 109)	Adults with gallstones, including acute cholecystitis	Retrospective; 2013–2019	NR	Any: conversion, time ≥120 min, gangrene/abscess (pathology), or severe adhesions complicating Calot dissection.	No overlap identified based on available information.
42 Suzuki et al. 2025 [7]	Japan	Five institutions; pooled multicenter dataset	1,898 (1,518 / 380)	Benign gallbladder disease, including acute cholecystitis; preoperative CT required	Retrospective multicenter; 2015–2020	Mixed disease indications; elective/emergency distribution NR	Failure to achieve CVS followed by open conversion, subtotal LC and/or fundus-first approach (262 bailouts).	No overlap identified based on available information.
43 Zhu et al. 2025 [12]	China	Shijiazhuang People's Hospital; one center	265 (185 / 80)	Benign gallbladder disease (acute/chronic cholecystitis or polyps)	Retrospective; September 2022–June 2024	Early and later LC recorded; urgency classification NR	Any: operative time ≥120 min, failure to achieve CVS within 40 min or open conversion.	No overlap identified based on available information.
44 Zhang et al. 2025 [6]	China	Two Shanghai hospitals for development; one prospective implementation site	9,414 + 835	Benign gallbladder conditions	Retrospective development (2013–2018); prospective implementation (2019–2021)	Scheduled LC; elective/emergency distribution NR	High-risk LC: conversion or complications (biliary/organ injury, bleeding, leak, infection and other events).	No overlap identified based on available information.
45 Wu et al. 2025 [13]	China	Eight centers; one development center and seven external centers	3,191 (1,658 / 1,533)	First acute calculous cholecystitis; 263 conversions	Retrospective multicenter cross-sectional; January 2013–December 2023	Early LC within 72 h of symptom onset	Conversion from LC to open surgery.	No overlap identified based on available information.
46 Naveed et al. 2025 [54]	Pakistan	Railway General Hospital, Rawalpindi; one center	220	Adults 18–70 with symptomatic gallbladder disease	Cross-sectional diagnostic validation; July 2024–January 2025	Emergency surgery excluded	Any: time >60 min, biliary leakage, cystic-duct/artery injury or conversion.	No overlap identified based on available information.

Study and reference	Country	Study setting	Sample size n	Clinical population	Study design	Elective or emergency status	Prediction target and outcome definition	Underlying cohort / possible cohort overlap
47 Teja et al. 2025 [55]	India	Sri Ramachandra University tertiary institute, Chennai; one center	222 (21 conversions)	Adult cholelithiasis; acute cholecystitis, recent ERCP and upper abdominal surgery excluded	Prospective; 2014–2016	Elective	Conversion to open cholecystectomy.	No overlap identified based on available information.
48 Megahed et al. 2025 [56]	Saudi Arabia	National Guard Hospital/King Abdulaziz Medical City, Riyadh; one tertiary hospital	530	Chronic calculous cholecystitis; adult predominance (all ages eligible)	Retrospective validation; October 2017–October 2023	Elective	Easy/difficult/very difficult based on operative time, spillage, injury and conversion.	No overlap identified based on available information.
49 Ahmed et al. 2025 [57]	Ethiopia	Yekatit 12 and St Paul's Millennium Medical College hospitals, Addis Ababa	200	Symptomatic cholelithiasis; resolved cholecystitis/pancreatitis allowed, active acute cholecystitis excluded	Prospective cross-sectional; August 2022–July 2024	Elective	DLC by Sugrue operative criteria (mild 0–2 versus greater difficulty); modified 16-point intraoperative grading also evaluated.	No overlap identified based on available information.
50 Soniya et al. 2026 [58]	India	SRM Medical College Hospital and Research Centre; one teaching hospital	50	Adults with symptomatic cholelithiasis/cholecystitis	Prospective observational; 12 months	Elective	Composite operative difficulty: >60 min, dense adhesions/frozen Calot triangle, difficult anatomy/dissection, spillage, injury, bleeding or conversion.	No overlap identified based on available information.
51 Moreno Delgado et al. 2026 [59]	Mexico	Three secondary/tertiary hospitals in Coahuila/Durango; pooled cohort	250	Gallstone disease; observed age 15–78 years	Retrospective case-control; February 2022–May 2025	Elective or early LC within 72 h for acute cholecystitis	Parkland grade ≥4 (85 difficult; 165 non-difficult).	No overlap identified based on available information.
52 Sudhir et al. 2026 [60]	India	JSS Medical College Hospital, Mysuru; one center	60	Adults with acute/chronic cholecystitis, with or without stones	Prospective observational; May 2023–November 2024	Elective	Easy ≤60 min without complications; difficult 61–120 min and/or minor spillage/injury without conversion; very difficult >120 min and/or conversion.	No overlap identified based on available information.

Study and reference	Country	Study setting	Sample size n	Clinical population	Study design	Elective or emergency status	Prediction target and outcome definition	Underlying cohort / possible cohort overlap
53 Yang et al. 2026 [14]	China	Three tertiary hospitals; Jinzhou development, two other hospitals validation	580 (406 / 174)	Acute cholecystitis after PTGBD	Retrospective multicenter; January 2013–June 2024	LC after stabilization following PTGBD; urgency classification NR	Difficult LC: conversion, operative time ≥120 min or blood loss ≥300 mL (wording of conversion criterion ambiguous).	No overlap identified based on available information.
54 Şenocak et al. 2026 [61]	Turkey	Gülhane Training and Research Hospital, Ankara; tertiary referral center	183 (33 conversions)	Adults with acute calculous cholecystitis	Retrospective analysis of prospective database; May 2020–May 2024	NR	Conversion from LC to open cholecystectomy.	No overlap identified based on available information.
55 Basukala et al. 2026 [62]	Nepal	Single tertiary institution (Shree Birendra Hospital)	405	Gallstone/biliary disease; observed age 17–80	Institutional cross-sectional study; January 2022–June 2023	21 emergency; 138 elective; 246 delayed	Nassar grades III–IV difficult versus I–II.	No overlap identified based on available information.
56 Kumar et al. 2026 [63]	India	Deen Dayal Upadhyay Hospital, New Delhi; one center	150	Adults with symptomatic cholelithiasis; acute cholecystitis/pancreatitis excluded	Prospective cohort; February 2021–July 2022	Elective	Easy/difficult/very difficult based on <60, 60–120, >120 min, spillage, duct/artery injury and conversion; difficult groups combined for ROC.	No overlap identified based on available information.
57 Nagarla et al. 2026 [64]	India	Dr D. Y. Patil Medical College Hospital, Pune; one center	150	Symptomatic cholelithiasis; age 21–80; acute cholecystitis excluded	Prospective observational; recruitment dates NR	Elective	Easy <60 min without spillage/injury; difficult 60–120 min and/or spillage/injury; very difficult >120 min or conversion.	No overlap identified based on available information.
58 Ayantunde et al. 2026 [65]	United Kingdom	Southend University Hospital, England; one NHS Trust site	353	Adults with gallstone disease; age 20–96	Prospective observational validation; February 2022–January 2025	340 delayed/elective; 13 early (within 7 days)	Nassar III–IV versus I–II operative difficulty.	No overlap identified based on available information.
59 Utsumi et al. 2026 [15]	Japan	National Hospital Organization Fukuyama Medical Center; one center	311 (223 / 88)	Acute cholecystitis; common bile duct stones/pancreatitis and prior upper abdominal surgery excluded	Retrospective cohort; January 2012–December 2024	Early LC routinely used; delayed LC after drainage in selected high-risk cases	Blood loss ≥50 mL, time ≥150 min or conversion (Methods uses > instead of ≥).	No overlap identified based on available information.

Panel B Predictors model type and validation

Study and reference	Predictor modalities and model inputs	Model or tool type	Development validation or comparison role	Internal validation	External temporal or independent validation
01 Kareem et al. 2010 [21]	US: wall >4 mm, pericholecystic fluid, severely contracted gallbladder, empyema and gallbladder filled with stones.	Five-finding US rule/count; no weighted model	Same-cohort assessment of imaging rule	None reported	None reported
02 Vyas et al. 2013 [20]	Clinical + US. RSCLO: sex, tenderness, upper abdominal surgery, wall ≥4 mm, age ≥60, cholecystitis. New rule: age, sex, prior acute attack, BMI ≥30, prior upper surgery, contracted gallbladder, wall ≥4 mm, stone >1.5 cm.	Existing RSCLO vs new eight-item equal-weight score	Independent validation of RSCLO plus development/apparent evaluation of new score	None reported	New-center cohort for existing RSCLO; none for new rule
03 Gupta et al. 2013 [22]	Clinical + US: age, sex, prior acute-cholecystitis admission, BMI, scar location, palpable gallbladder, wall thickness, fluid and impacted stone.	Existing 15-point Randhawa–Pujahari score	Independent validation of existing score	None reported	Independent new-center cohort
04 Joshi et al. 2015 [23]	Clinical + laboratory + US: Randhawa factors, four comorbidity items, contracted gallbladder, WBC, bilirubin, ALT, AST and ALP.	Modified point score; reported maximum 25	Development and apparent evaluation	None reported	None reported
05 Bourgouin et al. 2016 [4]	Clinical + laboratory: male sex, prior cholecystitis, neutrophil count, fibrinogen and ALP.	DiLC weighted point score; range 0–18	Development with internal resampling validation	Bootstrap 2,000 iterations of 400 patients each	None reported
06 Sutcliffe et al. 2016 [24]	Clinical + US: age, sex, ASA class, indication, thick-walled gallbladder and CBD dilation.	CLOC regression-derived point score; range 0–14	Development with internal split validation	Random 3:1 patient split; pooled hospitals	None in this publication
07 Beksac et al. 2016 [25]	Clinical + laboratory: male sex, age >55, ALP >80 IU/L and prior abdominal surgery.	Four-factor logistic probability model	Development and apparent evaluation	None reported	None reported
08 Siddiqui et al. 2017 [26]	US only: wall ≥4 mm, transverse diameter >5 cm, impacted stone, CBD >6 mm, fluid, >1 stone and liver span >15.5 cm.	Seven-item weighted US score; maximum 11	Development and apparent evaluation	None reported	None reported
09 Saleem & Abdallah 2018 [27]	Clinical + US: age, sex, prior acute-cholecystitis admission, BMI, scar location, palpable gallbladder, wall thickness, fluid and impacted stone.	Existing 15-point Randhawa–Pujahari score	Independent validation of existing score	None reported	Independent Egyptian cohort
10 Hu et al. 2018 [28]	Clinical + US: upper abdominal surgery, BMI >30, visible CBD stone, impacted neck stone and wall thickness (mm).	Existing Cairns Prediction Model (CPM), logistic nomogram	Independent external validation without recalibration	None reported	Townsville vs Cairns derivation hospital, 350 km apart
11 Veeranki & Togale 2018 [66]	Clinical + US: age, sex, prior acute-cholecystitis admission, BMI, scar location, palpable gallbladder, wall thickness, fluid and impacted stone.	Existing 15-point score as used by Gupta/Randhawa	Independent validation of existing score	None reported	Independent new-center cohort

Study and reference	Predictor modalities and model inputs	Model or tool type	Development validation or comparison role	Internal validation	External temporal or independent validation
12 Wennmacker et al. 2019 [29]	Clinical + laboratory + US: acute-cholecystitis diagnosis, CRP >10.5 mg/L, pericholecystic fluid.	Three-factor regression-derived point score; maximum 4.5	Development and apparent evaluation	None reported	None reported
13 AbdelDayem et al. 2020 [30]	Clinical + US/procedure history: age >62, wall >3 mm, other acute inflammatory signs, large gallstone, pancreatitis, need for ERCP/MRCP.	Six-component weighted point score; maximum 8	Development and apparent evaluation	None reported	None reported
14 Boraii & Abdelaziz 2020 [31]	Clinical + US: age, sex, prior acute-cholecystitis admission, BMI, scar location, palpable gallbladder, wall thickness, fluid and impacted stone.	Existing 15-point Randhawa–Pujahari score	Independent score validation	None reported	Independent Egyptian cohort
15 Nassar et al. 2020 [5]	Clinical + US: age, sex, ASA class, biliary diagnosis, wall ≥3 mm, CBD >6 mm, prior ERCP and admission type.	Regression-derived 0–19 point risk score	Development with internal and external validation	Random 3:1 split of CholeS cohort	Separate reference cohort (geographic/service validation)
16 Kumar et al. 2021 [32]	Clinical + US: age, sex, prior acute-cholecystitis admission, BMI, scar location, palpable gallbladder, wall thickness, fluid and impacted stone.	Existing 15-point Randhawa–Pujahari score	Independent validation of existing score	None reported	Independent new-center cohort; local ROC cutoffs explored
17 Goyal et al. 2021 [33]	Clinical + US: age, sex, admission history, ERCP, diabetes, BMI, prior abdominal surgery, palpable gallbladder, wall thickness, fluid, impacted stone and contracted gallbladder.	New modified 18-point score compared with existing Randhawa score	Development/apparent evaluation; concurrent independent validation of original score	None reported	New-center cohort for comparator; none for new score
18 Wibowo et al. 2022 [34]	Clinical + laboratory + US: age, sex, acute-cholecystitis admission, BMI, scar, palpable GB, wall thickness, WBC ≥10,000/μL.	Adapted eight-item point score; maximum 15	Local adaptation/evaluation of previous scoring approach	None reported	None for adapted score; same-cohort cutoff selection
19 Matsumoto et al. 2022 [35]	Clinical + CT: symptom-onset-to-PTGBD ≥3 days and maximal GB wall ≥10 mm (measured just before PTGBD); one point each.	Two-factor 0–2 point score	Development and separate-cohort validation	None reported	Temporal locally plus geographic second-hospital cohort (65 total)
20 Ramírez-Giraldo et al. 2022 [36]	Clinical + US (Nassar): age, sex, ASA class, primary diagnosis, wall thickness, CBD diameter, ERCP and admission type.	Existing Nassar preoperative 0–19 point scale	Independent validation	None reported	Independent Colombian cohort of published UK score
21 Reddy et al. 2022 [37]	Clinical + US: age, sex, prior acute-cholecystitis admission, BMI, scar location, palpable gallbladder, wall thickness, fluid and impacted stone.	Established 15-point Randhawa-style score	Independent new-cohort score evaluation; locally selected thresholds	None reported	Independent hospital cohort; same-cohort threshold selection
22 Paul et al. 2022 [38]	Clinical + US: age, sex, previous attacks of cholecystitis, BMI, scar location, palpable gallbladder, wall thickness, fluid and impacted stone.	Existing Randhawa 15-point score	Independent validation and risk-factor analysis	None reported	Independent acute-cholecystitis cohort

Study and reference	Predictor modalities and model inputs	Model or tool type	Development validation or comparison role	Internal validation	External temporal or independent validation
23 Chen et al. 2022 [39]	Clinical + laboratory + imaging: BMI >25, WBC >10×10⁹/L, impacted neck stone, >4 acute attacks in prior 2 months, wall >5 mm, largest stone >2 cm.	Six-variable logistic probability model	Development and apparent evaluation	None reported	None reported
24 Kotb et al. 2023 [40]	Clinical + US: age >50, BMI >25, prior upper abdominal surgery, acute-cholecystitis history, thick gallbladder wall.	Regression-derived point score; maximum 25	Development and apparent evaluation	None reported	None reported
25 Mazni et al. 2022 [41]	Clinical + US: age, sex, operative indication, ASA class, wall thickness and CBD dilation (CLOC).	Existing CLOC point score; secondary logistic risk-factor analysis	Independent geographic validation of CLOC	None reported	Independent Indonesian hospital cohort
26 Morsy et al. 2023 [42]	Clinical + US: age, sex, prior acute-cholecystitis admission, BMI, scar location, palpable gallbladder, wall thickness, fluid and impacted stone. Prior surgery also included in hospitalization item; wall-thickness categories modified.	Adapted Randhawa-style 15-point score	Same-cohort evaluation of adapted score	None reported	None reported
27 Kapoor et al. 2023 [8]	Advanced US: ARFI/VTI pericholecystic stiffness at fundus/body/neck, extent/grade of adhesions and contracted GB. Conventional clinical/US score comparator.	Adhesion detection/staging (ADS); point-score comparator	ADS development/apparent evaluation; comparison with modified Randhawa	None reported	None for ADS; independent evaluation of modified comparator
28 Trehan et al. 2023 [43]	Clinical + US: age, sex, prior acute-cholecystitis admission, BMI, scar location, palpable gallbladder, wall thickness, fluid and impacted stone.	Existing Randhawa–Pujahari 15-point score	Independent validation	None reported	Independent Indian cohort
29 Lattoo et al. 2023 [44]	Clinical + US: age, sex, prior acute-cholecystitis admission, BMI, scar location, palpable gallbladder, wall thickness, fluid and impacted stone.	Existing Randhawa–Pujahari 15-point score	Independent validation in rural setting	None reported	Independent rural hospital cohort
30 Ramírez-Giraldo et al. 2023 [45]	Clinical + laboratory + US across seven scores: age/sex, BMI, biliary inflammation/procedures, tenderness, scar/surgery, ASA/admission type, WBC/ALP, GB wall/size, stones/fluid, CBD/liver size.	Seven existing scores: Randhawa, Siddiqui, Kama, Tongyoo, Carrizo, Nassar, Alponat	Independent same-population score comparison	None reported	Independent Colombian evaluation of existing models
31 Mishima et al. 2024 [46]	Clinical + laboratory + imaging: CRP bands ≥5/10/15 mg/dL (1/2/3 points); symptoms >72 h (1); TG18 grade-II local imaging inflammation (1).	Regression-derived 0–5 point score	Development and apparent comparison with TG18	None reported	None reported

Study and reference	Predictor modalities and model inputs	Model or tool type	Development validation or comparison role	Internal validation	External temporal or independent validation
32 Serrano-González et al. 2024 [47]	Clinical + laboratory + US (Labbad–Vivas): sex, age, diabetes, BMI, prior biliary events/ERCP, abdominal surgery, WBC, wall thickness, palpable GB, impacted stone, fluid, cirrhosis.	Existing 12-item Labbad–Vivas point score	Independent validation against Parkland; same-cohort PGS regression	None reported	Independent hospital evaluation of previously developed score
33 Stoica et al. 2024 [48]	New model: laboratory fibrinogen + US wall thickness and transverse diameter. Tongyoo/Randhawa comparator: age, sex, biliary inflammation, BMI, palpable GB, scar, wall, fluid, impaction.	Existing point-score evaluation plus new logistic model	Independent comparator evaluation; same-cohort model development	None reported for new model	Independent institutional evaluation of existing score; none for new model
34 Acharya et al. 2024 [49]	Clinical + US: age, sex, prior GB-related hospitalization, BMI, scar, palpable GB, wall thickness, fluid and impacted stone.	Existing 15-point score labeled RSCLO by authors	Independent score validation	None reported	Independent Nepalese cohort
35 Wei et al. 2024 [9]	Laboratory + CT: CRP >8.2 mg/L, pericholecystic effusion, contact between GB and stomach/duodenum.	Logistic-regression nomogram	Development and apparent evaluation	None reported; calibration curve shown in same cohort	None reported
36 Martínez-Mier et al. 2024 [50]	Clinical + US (Nassar/CholeS): age, sex, ASA class, biliary diagnosis, thick GB wall, CBD dilation, ERCP and admission type.	Existing 0–19 point score	Independent validation of Nassar/CholeS score	None reported	Independent Mexican cohort
37 Khan et al. 2024 [51]	US-only modified Siddiqui tool described as a six-point chart; individual items/weights not reproduced. Clinical/laboratory measures collected for comparison.	Modified existing US score	Independent evaluation of modified US tool	None reported	Independent tertiary-center cohort; score modified
38 Sun et al. 2025 [10]	Clinical + laboratory + US + CT radiomics: acute/chronic disease, fibrinogen, CRP, wall thickness, short GB diameter, PLR, apoA-1, ALP, LDL-C and Rad-Score (9 radiomics features).	Six ML algorithms; selected combined random forest	Development and internal validation; model comparison	70:30 split (1,439/616); 10-fold CV	None; same-center holdout, despite external wording in text
39 Agrawal et al. 2025 [52]	Preoperative clinical/history, laboratory and US scores (POS). Training links POS to operative-note/video action scores using co-occurrence matrices; full preoperative input definitions NR.	AI/co-occurrence-matrix score model	Pilot development and internal holdout evaluation	First 17 training; next 8 test (same cohort)	None reported
40 Zia et al. 2025 [11]	Clinical + US (Randhawa): age, sex, prior acute-cholecystitis admission, BMI, scar, palpable gallbladder, wall thickness, fluid, impacted stone.	Existing 15-point score	Independent validation of Randhawa score	None reported	Independent new-center cohort in Pakistan
41 Huang et al. 2025 [53]	Clinical + laboratory + US: age ≥53, CAR ≥0.108, NLR ≥3.479, WBC ≥8.055, liver-function score (TBIL/ALP/AST), wall >2 mm and Calot-triangle clarity.	Logistic nomogram with LASSO/SVM/tree analyses	Development and internal validation	70:30 split (253/109); CV used in feature selection	None reported

Study and reference	Predictor modalities and model inputs	Model or tool type	Development validation or comparison role	Internal validation	External temporal or independent validation
42 Suzuki et al. 2025 [7]	Clinical + laboratory + imaging: age, sex, gallbladder drainage, prior endoscopic sphincterotomy, TG18 acute-cholecystitis grade, impacted neck stone, serum CRP.	Logistic-regression nomogram	Development and internal validation; surgeon-expertise comparison	Random 4:1 split of pooled data (1,518/380)	None; hospitals pooled before random split
43 Zhu et al. 2025 [12]	Conventional imaging: wall thickness, impacted GB neck stone, cystic-duct length and diameter (MRCP; averaged radiologist readings).	Logistic-regression nomogram	Development and internal validation	Random 70:30 split; 10-fold CV for LASSO selection	None reported
44 Zhang et al. 2025 [6]	Clinical + US + surgeon experience: temperature ≥38.5°C, attacks ≥3, wall ≥5 mm, impacted neck stone, CBD ≥8 mm; surgeon case-volume strata.	Regression-derived point score and management pathway	Development and prospective implementation evaluation	Apparent ROC evaluation; no resampling/split reported	Later prospective cohort (n=835); management changed according to score, limiting pure predictive validation
45 Wu et al. 2025 [13]	Clinical + laboratory + US: CRP, ASA class, stone location (neck/body), TG18 grade and surgeon seniority (≤5, >5–10, >10 years).	Logistic-regression nomogram; ML comparators	Development and geographic external validation	10-fold CV for LASSO feature selection	Seven separate centers (n=1,533)
46 Naveed et al. 2025 [54]	US only: wall ≥4 mm, transverse diameter ≥5 cm, impacted stone, CBD >6 mm, pericholecystic fluid, >1 stone, liver ≥15.5 cm.	Existing seven-item US score; maximum 11	Independent validation of Siddiqui US score	None reported	Independent new-center cohort
47 Teja et al. 2025 [55]	Final score: US wall thickness >4 mm and contracted gallbladder (one point each). Clinical/laboratory candidates not retained.	Two-factor US point score	Development and apparent evaluation	None reported	None reported
48 Megahed et al. 2025 [56]	Clinical + US: age, sex, prior admission, BMI, scar location, palpable gallbladder, wall thickness, impacted stone and fluid.	Modified Randhawa 15-point score	Independent validation of existing score	None reported	Independent Saudi institutional cohort
49 Ahmed et al. 2025 [57]	Clinical + US: age, sex, prior acute-cholecystitis admission, BMI, palpable GB, abdominal scar, impacted stone. Intraoperative variables belong to separate score/secondary analysis.	Adapted preoperative point score and intraoperative comparator	Evaluation of modified existing preoperative score in new setting	None reported	Independent setting, but rule adapted and evaluated in same cohort
50 Soniya et al. 2026 [58]	US: wall thickness, gallbladder distension, impacted/non-mobile stone, CBD diameter, multiple stones, pericholecystic fluid, liver span.	US point score (maximum 11)	Development and apparent evaluation	None reported	None reported
51 Moreno Delgado et al. 2026 [59]	Clinical + laboratory: age ≥50, male sex, NLR ≥2, indirect bilirubin ≥1.2 mg/dL, type 2 diabetes, SII ≥400.	Regression-derived point score; maximum 15	Model development with internal validation	5-fold CV; 1,000 bootstrap samples	None; three hospitals pooled for development
52 Sudhir et al. 2026 [60]	Clinical + laboratory + US: age, sex, pain duration, number of attacks, scar, diabetes, WBC, HbA1c, wall thickness, pericholecystic fluid, stone size/number/impaction.	13-variable point score; maximum 15	Development and apparent evaluation	None reported	None reported

Study and reference	Predictor modalities and model inputs	Model or tool type	Development validation or comparison role	Internal validation	External temporal or independent validation
53 Yang et al. 2026 [14]	Clinical + laboratory + US: onset-to-PTGBD interval, upper abdominal surgery, WBC, gallstones, wall thickness, neutrophil percentage, PTGBD-to-LC interval.	Eight ML algorithms; selected naive Bayes	Development and geographic external validation	10-fold CV (mean NB AUC 0.837)	Two other hospitals, n=174
54 Şenocak et al. 2026 [61]	Clinical + laboratory + US: age, diabetes, WBC, LDH, wall thickness. TG18 added in hybrid model/comparisons.	Firth logistic models; derived 14-point conversion score	Development and internal validation; TG18 comparison	Bootstrap optimism correction	None reported
55 Basukala et al. 2026 [62]	Clinical + US: demographic/ASA data, admission type, biliary diagnosis, wall thickness, CBD dilation, ERCP. Complete 0–19 score form referenced in supplement.	Existing preoperative difficulty score; secondary logistic regression	Independent score evaluation; same-cohort risk-factor analysis	None reported	Independent institutional cohort for existing score
56 Kumar et al. 2026 [63]	Clinical + US: age, sex, prior admission, BMI, scar, palpable gallbladder, wall thickness, impacted stone, pericholecystic fluid.	Modified Randhawa–Pujahari point score	Independent validation of an existing score with modified weight	None reported	Independent new-center cohort; score modification noted
57 Nagarla et al. 2026 [64]	Clinical + laboratory + US, 15 items: age, sex, cholecystitis, diabetes, ERCP, BMI, scar, ALP, wall thickness, impacted/large stone, contracted gallbladder, fluid, CBD diameter, procedure for acute cholecystitis.	15-item point score (one point per item)	Same-cohort score evaluation	None reported	None reported
58 Ayantunde et al. 2026 [65]	Clinical + laboratory + conventional imaging: demographics, BMI, comorbidities/ASA, liver-function abnormalities, gallstone complications and imaging findings. Complete weighted items in Online Appendix 1.	Traffic-light preoperative point score	Validation during routine implementation	None reported	New hospital within the same Trust as development site (Broomfield)
59 Utsumi et al. 2026 [15]	Clinical + laboratory + CT: male sex, GPS ≥1 (CRP/albumin), NLR ≥6.75, symptom duration ≥72 h, pericholecystic fat hyperdensity.	Logistic-regression nomogram	Development and internal validation	Stratified random 70:30 split (223/88); 1,000-bootstrap calibration	None reported

Panel C Performance reporting and clinical translation

Study and reference	Discrimination AUC and threshold performance	Calibration	DCA or utility and explainability	Complete model specification availability	Clinical implementation or impact	Key notes and limitations
01 Kareem et al. 2010 [21]	AUC: NR. Wall >4 mm: sensitivity 69%, specificity 96.5%; fluid specificity 100%. Abstract claims 100% accuracy for >2 findings; Table 4 reports 78.5% for three findings.	NR	DCA/utility: NR Explainability: No formal analysis reported	Five ancillary findings and count categories described; contracted/empyema definitions incomplete.	Clinical use or impact not evaluated	Tables/abstract contain contradictory diagnostic estimates; denominators mix difficult LC and conversion. Includes age 16. Twenty-nine difficult procedures and seven conversions.

Study and reference	Discrimination AUC and threshold performance	Calibration	DCA or utility and explainability	Complete model specification availability	Clinical implementation or impact	Key notes and limitations
02 Vyas et al. 2013 [20]	AUC and sensitivity/specificity: NR. RSCLO: 17 moderate/severe cases all difficult/converted; 8/83 mild cases difficult. New rule: 10/11 moderate cases difficult/converted; 15/89 mild cases difficult.	NR	DCA/utility: NR Explainability: No formal analysis reported	RSCLO coefficients/constant −20 in Table 1; full new-score points in Table 2 and bands 1–3/4–6/7–8 in Methods.	Clinical use or impact not evaluated	Three conversions. Existing-score validation omitted from manuscript Section 3.5 ref. list; harmonize with ref. 20. Comparison also develops a new score. Stone-size equality and score-zero handling NR.
03 Gupta et al. 2013 [22]	AUC 0.86. At score 5: reported sensitivity 95.74%, specificity 73.68%; PPV easy 90%, difficult 88%.	NR	DCA/utility: NR Explainability: No formal analysis reported	Complete nine-factor point allocations/bands in Table 1.	Clinical use or impact not evaluated	Reported sensitivity/specificity do not align with Table 5 counts. Conversion stated as 4.28% in abstract/text but 12/210 in tables/results; source discrepancy retained.
04 Joshi et al. 2015 [23]	AUC 0.779 (95% CI 0.657–0.883). Score ≥6: sensitivity 53.8%, specificity 89.2%, PPV 63.64%, NPV 84.62% (abstract/table).	NR	DCA/utility: NR Explainability: No formal analysis reported	Point table and bands provided; ALT item maximum 1 but positive weight 2, so total/coding require clarification.	Clinical use or impact not evaluated	Surgeons blinded to preoperative score. Includes age 14. Results prose swaps PPV/NPV; abstract/table retained. Operative-time categories have a 91–120 min gap.
05 Bourgouin et al. 2016 [4]	Apparent AUC 0.80; bootstrap-corrected AUC 0.76. Cutoff 4: sensitivity 65.2%, specificity 81.1%, PPV 65.2%, NPV 81.1%, accuracy 75.5%.	NR	DCA/utility: NR Explainability: No formal analysis reported	Full five-factor point table and thresholds in Table 3; probability intercept NR.	Clinical use or impact not evaluated	Analyzed sample is 420, distinct from abstract's 644 screened. Clinical-use benefits proposed; outcome correlations are not a clinical-impact evaluation.
06 Sutcliffe et al. 2016 [24]	Development logistic AUC 0.811, simplified score 0.802; internal score AUC 0.766 (SE 0.027). Score >6: sensitivity 77.1%, specificity 65.4%; conversion 7.1% vs 1.2%.	Formal calibration metrics: NR; observed conversion by score with pooled fitted reference curve.	DCA/utility: NR Explainability: No formal analysis reported	Full six-factor points in Table 5; original probability-model intercept NR; US binary thresholds not explicitly defined.	Clinical use or impact not evaluated	Multicenter random split is internal validation. Cutoff selected in validation set. Shares CholeS source cohort with Nassar 2020.
07 Beksac et al. 2016 [25]	AUC 0.77 (95% CI 0.71–0.84). Probability >0.5: sensitivity 70%, specificity 79%.	NR	DCA/utility: NR Explainability: No formal analysis reported	Complete coefficients and intercept −2.495 in Table 2; binary coding/formula illustrated.	Clinical use or impact not evaluated	50% conversion prevalence in sampled analysis vs 7.7% in source cohort; predicted absolute risks need prevalence correction before transport. Ultrasound candidates not retained in final model. Uploaded PDF is author proof.
08 Siddiqui et al. 2017 [26]	AUC: NR. Score >5: sensitivity 80.7%, specificity 91.7%; reported predictive values difficult 78.8%, easy 92.6%.	NR	DCA/utility: NR Explainability: No formal analysis reported	Complete point rule in Table 1 (2/2/2/2/1/1/1), cutoff >5.	Clinical use or impact not evaluated	Abstract counts (73 predicted difficult) differ from Table 3 (84). Sensitivity 80.7% does not match 66/83; source estimates retained. Surgical difficulty is primary composite, not conversion alone.

Study and reference	Discrimination AUC and threshold performance	Calibration	DCA or utility and explainability	Complete model specification availability	Clinical implementation or impact	Key notes and limitations
09 Saleem & Abdallah 2018 [27]	AUC 0.86 (95% CI 0.77–0.92). Score >5: sensitivity 74.3%, specificity 96.9%, PPV 92.9%, NPV 87.5%, accuracy 85.6% (reported).	NR	DCA/utility: NR Explainability: No formal analysis reported	Full point allocations and bands in Tables 1–2.	Clinical use or impact not evaluated	Twelve conversions. Secondary logistic associations do not constitute a separately validated preoperative prediction tool.
10 Hu et al. 2018 [28]	External AUC 0.87 (95% CI 0.82–0.93); threshold sensitivity/specificity NR.	NR	DCA/utility: NR Explainability: No formal analysis reported	Complete five coefficients, intercept −9.36 and input coding reproduced in Methods.	Routine use at original Cairns site for three years stated; no measured clinical-impact evaluation.	Original coefficients used unchanged. Broad age eligibility includes pediatric patients; manuscript adult-population criterion requires review. Uploaded version is accepted manuscript.
11 Veeranki & Togale 2018 [66]	AUC: NR. For EASY classification: sensitivity 86.36%, specificity 75%; PPV easy 90.48%, difficult 66.67%.	NR	DCA/utility: NR Explainability: No formal analysis reported	Full nine-factor point rule in Table 1; operative grading in Table 2.	Clinical use or impact not evaluated	Eight difficult/very-difficult cases. Sensitivity/specificity refer to easy, not difficult, as clarified in Results. Methods text risk-band typo differs from Table 4.
12 Wennmacker et al. 2019 [29]	AUC 0.86 (95% CI 0.80–0.91). Score ≥2.5: sensitivity 77.7%, specificity 81.7%, PPV 87.0%, NPV 69.9%.	NR	DCA/utility: NR Explainability: No formal analysis reported	Complete point rule in abstract: diagnosis 2, CRP 1.5, fluid 1; probability intercept NR.	Clinical use or impact not evaluated	No held-out or resampled validation. Additional baseline details/ROC figures cited in unprovided supplements; main scoring rule is available.
13 AbdelDayem et al. 2020 [30]	AUC: NR. Score ≥2 for prolonged time: sensitivity 46%, specificity 70%. Score ≥4 for conversion-associated difficulty: sensitivity 73%, specificity 87%.	NR	DCA/utility: NR Explainability: No formal analysis reported	Weights 2/2/1/1/1/1 and risk bands in Table 6; large-stone threshold and some inflammatory-sign definitions NR.	Clinical use or impact not evaluated	Includes children (minimum age 10); manuscript adult-population eligibility requires review. Online 2019, issue 2020.
14 Boraii & Abdelaziz 2020 [31]	AUC 0.902. Cutoff 7: sensitivity 77.8%, specificity 86.3%; cutoff 5: sensitivity 88.9%, specificity 68.6%.	NR	DCA/utility: NR Explainability: No formal analysis reported	Variables and bands given; individual original point allocations not fully tabulated.	Clinical use or impact not evaluated	Nine difficult/very-difficult cases; five conversions. Source wording says items scored 0/1/2 despite score's 4-point admission item; local application cannot be fully reconstructed.
15 Nassar et al. 2020 [5]	Internal AUC 0.764 (95% CI 0.741–0.786); external AUC 0.789 (0.773–0.806). External difficult rates: 11.0% at score 0–1, 31.1% at 2–6, 80.0% at ≥7.	NR	DCA/utility: NR Explainability: No formal analysis reported	Complete eight-factor point allocations in Table 4; risk categories 0–1/2–6/≥7.	Clinical use or impact not evaluated	Cohort totals exceed complete-case model samples. Shares CholeS source data with Sutcliffe 2016; publications are not independent patient cohorts. Online 2019, manuscript issue year 2020.
16 Kumar et al. 2021 [32]	Difficult/very difficult: AUC 0.974 (95% CI 0.95–0.99), cutoff 5.5, sensitivity 96.9%, specificity 97.3%. Very difficult vs difficult: AUC 0.782, cutoff 8.5.	NR	DCA/utility: NR Explainability: No formal analysis reported	Point weights and risk bands in Table 2; some categories combined for presentation.	Clinical use or impact not evaluated	Original S1 apparent-evaluation label corrected to independent score validation, consistent with manuscript ref. 32. Includes age 14. Duplicate Kumar PDF is the same article.

Study and reference	Discrimination<br>AUC and threshold performance	Calibration	DCA or utility and explainability	Complete model specification availability	Clinical implementation or impact	Key notes and limitations
17 Goyal et al. 2021 [33]	Figure 1: modified AUC 0.945; Randhawa AUC 0.874. Both sensitivity 95%; specificity 92% vs 76%; modified accuracy 95.1%. Text also reports regression ROC AUC 0.97.	NR	DCA/utility: NR Explainability: No formal analysis reported	12-item weights and risk bands in Tables 1–2; exact boundary handling and contracted-gallbladder definition incomplete.	Clinical use or impact not evaluated	New score and original-score validation distinguished. PPV differs between abstract and Results; reported ROC 0.97 concerns multivariable analysis, distinct from plotted score AUCs.
18 Wibowo et al. 2022 [34]	Easy/difficult AUC 0.849; cutoff 3.5: sensitivity 72.6%, specificity 87.5%. Difficult/very difficult AUC 0.779; cutoff 7.5: 70%/84.5%.	NR	DCA/utility: NR Explainability: No formal analysis reported	Full point table provided; hospital/BMI weights altered and WBC added.	Clinical use or impact not evaluated	Time-only outcome limits comparison with anatomical/safety targets. Title says five-year study; dates span six calendar years.
19 Matsumoto et al.<br>2022 [35]	Validation score 2: sensitivity 75.0%, specificity 98.1%. Individual development AUCs 0.795/0.854; composite score AUC NR.	NR	DCA/utility: NR Explainability: No formal analysis reported	Complete two-factor rule and thresholds provided.	Clinical use or impact not evaluated	Separate validation stronger than random split; only 12 bailout events per LC cohort. Initial-open comparisons are retrospective, not a score-guided impact trial.
20 Ramírez-Giraldo et al. 2022 [36]	AUC 0.88 (95% CI 0.85–0.92). Cutoff 8: sensitivity 75.15%, specificity 88.31%, PPV 87.32%, NPV 76.83%.	NR	DCA/utility: NR Explainability: No formal analysis reported	Complete point table and risk categories reproduced (Table 2).	Clinical use or impact not evaluated	Predictive scores calculated retrospectively; surgeon unaware. Text has isolated grade-number typos; explicit Table 1 uses III–IV.
21 Reddy et al. 2022 [37]	AUC 0.962 (95% CI 0.883–0.993); sensitivity 88.9%, specificity 92.3%; reported accuracy 86.36%.	NR	DCA/utility: NR Explainability: No formal analysis reported	Full 15-point rule displayed in Figure 1.	Clinical use or impact not evaluated	Abstract n=70 versus Results n=66. Existing rule confirmed in Figure 1; revise apparent-only classification in earlier S1 and harmonize manuscript Section 3.5.
22 Paul et al. 2022 [38]	AUC: NR. Sensitivity 82.6%, specificity 63.5%; conversion 6.9%.	NR	DCA/utility: NR Explainability: No formal analysis reported	Complete point table and risk categories (Tables 1–2).	Clinical use or impact not evaluated	Independent validation confirmed; manuscript Section 3.5 should include reference 38. Predictor associations do not constitute development of a separate validated model.
23 Chen et al. 2022 [39]	AUC 0.879 (95% CI 0.822–0.933). Probability cutoff 0.686: sensitivity 94.3%, specificity 52%.	Hosmer–Lemeshow χ^2=1.457, df=5, P>0.05 (development data).	DCA/utility: NR Explainability: No formal analysis reported	Complete coefficients, intercept −3.426 and binary coding in equation/Tables IV–V.	Clinical use or impact not evaluated	No resampling or held-out evaluation reported. Gallbladder puncture considered during fitting but absent from final equation.
24 Kotb et al. 2023 [40]	AUC 0.88. Score >13: sensitivity 70%, specificity 90%, accuracy 82%.	NR	DCA/utility: NR Explainability: No formal analysis reported	Five nonzero weights 5/11/3/2/4 in Table 2; thick-wall threshold NR; probability intercept NR.	Clinical use or impact not evaluated	Online publication 5 April 2023; journal issue is July–September 2022. Publication year updated to 2023 to match manuscript reference 40. Laboratory candidates not retained in final point score.

Study and reference	Discrimination AUC and threshold performance	Calibration	DCA or utility and explainability	Complete model specification availability	Clinical implementation or impact	Key notes and limitations
25 Mazni et al. 2022 [41]	AUC 0.788 (95% CI 0.582–0.994). Cutoff 6.5 (score ≥7): sensitivity 80%, specificity 79.1%.	NR	DCA/utility: NR Explainability: No formal analysis reported	CLOC source cited; point allocations not reproduced in main text. Secondary one-variable regression equation available in Table 3.	Clinical use or impact not evaluated	Only five conversions; wide CI. Original score assessed; locally optimized cutoff also reported. Figure CI upper limit 1.000 differs from main-text 0.994.
26 Morsy et al. 2023 [42]	AUC 0.972. Cutoff 5: sensitivity 84.2%, specificity 96.1%, PPV 95.2%, NPV 100%, accuracy 99.1%, as reported.	NR	DCA/utility: NR Explainability: No formal analysis reported	Point table provided; wall-thickness units printed as cm and history categories unclear.	Clinical use or impact not evaluated	Numerous table/count inconsistencies; accuracy/NPV do not reconcile with other diagnostic indices. Reported values retained.
27 Kapoor et al. 2023 [8]	ADS AUC 1.0 in abstract versus 0.91 in Results; sensitivity 91%, specificity 100%, NPV 93.1%, accuracy 96%. CSS AUC 0.63.	NR	DCA/utility: NR Explainability: No formal analysis reported	ADS staging rule and elastographic acquisition described in Table 1; CSS table includes ambiguous age weights.	Clinical use or impact not evaluated	Discrepant ADS AUCs/counts retained. Associations with time/LOS do not demonstrate a model-guided impact benefit.
28 Trehan et al. 2023 [43]	AUC 0.935 (95% CI 0.888–0.983); score >5: sensitivity 93.02%, specificity 83.12%.	NR	DCA/utility: NR Explainability: No formal analysis reported	Full point table and cutoff provided (Table 1).	Clinical use or impact not evaluated	Easy/difficult labels reversed in Table 4 compared with text and Table 5; ROC/2×2 findings reported separately.
29 Lattoo et al. 2023 [44]	AUC: NR. Reported sensitivity 86.41%, specificity 79.76%, PPV 92.51%, NPV 67%.	NR	DCA/utility: NR Explainability: No formal analysis reported	Full scoring weights and risk bands reproduced (Table 1).	Clinical use or impact not evaluated	Tables 4–5 give conflicting intraoperative counts; diagnostic indices should be interpreted cautiously. Issue 2023, online January 2024.
30 Ramírez-Giraldo et al. 2023 [45]	AUCs: Randhawa 0.747; Siddiqui 0.703; Kama 0.736; Tongyoo 0.783 (95% CI 0.745–0.822); Carrizo 0.740; Nassar 0.768; Alponat 0.761.	NR	DCA/utility: NR Explainability: No formal analysis reported	Rules/weights/formulas reproduced in Table 1; some item coding/typographic inconsistencies remain.	Clinical use or impact not evaluated	Surgeons blinded to retrospectively calculated scores. Cohort spans same institution/period as 2022 validation; potential sample overlap.
31 Mishima et al. 2024 [46]	AUC 0.724 in Results (0.721 abstract) versus TG18 0.609. Cutoff 3: sensitivity 68.8%, specificity 69.9%.	NR	DCA/utility: NR Explainability: No formal analysis	Complete point rule and probability equation in main text (p.3).	Clinical use or impact not evaluated	No open conversions in cohort; outcome is non-CVS. AUC differs slightly between abstract and Results; collection design not explicitly labeled.
32 Serrano-González et al. 2024 [47]	AUC 0.897 (95% CI 0.792–1.003, reported). LVS ≥16: sensitivity 80%, specificity 92%, PPV 85.7%, NPV 88.5%.	NR	DCA/utility: NR Explainability: No formal analysis	Full point table provided; Google Sheets calculator described.	Calculator provided; no evaluated care/impact study	Table assigns cirrhosis absent/present 3/2 points; verify apparent reversal. CI upper bound exceeds 1; small convenience sample.

Study and reference	Discrimination AUC and threshold performance	Calibration	DCA or utility and explainability	Complete model specification availability	Clinical implementation or impact	Key notes and limitations
33 Stoica et al. 2024 [48]	New model AUC 0.802 (probability >0.5: sensitivity 60.5%, specificity 86.6%). Tongyoo AUC 0.857 (>4: 67.6%/88%; abstract 0.856).	NR	DCA/utility: NR Explainability: No formal analysis	New model: exponentiated coefficients/intercept and units in Table 5; comparator point rule described.	Clinical use or impact not evaluated	New model performance is apparent. CRP unavailable in emergency setting and not used. Comparator cutoff selected in current cohort.
34 Acharya et al. 2024 [49]	AUC 0.922 (95% CI 0.865–0.980). Score >6: sensitivity 94.6%, specificity 90%, as reported.	NR	DCA/utility: NR Explainability: No formal analysis	Variables, maximum and cutoff described; complete point allocation not reproduced.	Clinical use or impact not evaluated	Table 4 (TP35/FP2/FN8/TN71) does not match reported sensitivity/specificity. No conversions/injuries; cutoff boundaries leave scores 5–6 unclear.
35 Wei et al. 2024 [9]	AUC 0.860 (individual predictors 0.612/0.739/0.644).	Calibration curve reported	DCA/utility: DCA reported Explainability: No formal analysis	Nomogram, ORs and predictor definitions provided; full intercept/code NR.	Clinical use or impact not evaluated	Calibration/DCA do not establish validation on independent patients; only one source cohort.
36 Martínez-Mier et al. 2024 [50]	Nassar target AUC 0.735 (95% CI 0.687–0.779); score >1.5: sensitivity 83.7%, specificity 50.8%. Outcome target AUC 0.743 (0.695–0.787).	NR	DCA/utility: NR Explainability: No formal analysis reported	Full point table and risk bands provided (Table 1).	Clinical use or impact not evaluated	Table prints CBD dilation <6 mm; this conflicts with expected dilation direction. Results include emergencies despite elective wording in flow description.
37 Khan et al. 2024 [51]	AUC: NR. Kappa −0.127 (P=0.275); only 1/16 difficult operations predicted as difficult.	NR	DCA/utility: NR Explainability: No formal analysis reported	Incomplete: scoring chart, full item definitions, weights and cutoff NR.	Clinical use or impact not evaluated	Poor agreement; clinical/laboratory associations are not inputs of the evaluated US-only tool.
38 Sun et al. 2025 [10]	Combined RF AUC 0.938/0.874; accuracy 0.868/0.811; Brier 0.106/0.146 (training/holdout).	Calibration curves and Brier scores	DCA/utility: DCA in training and holdout Explainability: SHAP	Rad-Score formula/preprocessing described and online calculator reported; trained RF object and complete code NR.	Calculator developed; no evaluated care/impact study	Postoperative drainage/LOS contribute to outcome. Same-center 70:30 split is internal validation. Exact split counts verified in Figure 2.
39 Agrawal et al. 2025 [52]	AUC: NR. Case-level predicted categories shown; no aggregate sensitivity/specificity or validation accuracy reported.	NR	DCA/utility: NR Explainability: Correlation matrices/workflow illustrated; no formal SHAP analysis	Partial: workflow/example matrices; complete POS weights and trained mapping/code NR.	Clinical use or impact not evaluated	Figure 2 describes generation of operative scores from preoperative data; training includes operative/video labels. Complete preoperative inference pipeline insufficiently specified; eligibility should be reviewed without assuming training labels are inference inputs.
40 Zia et al. 2025 [11]	AUC: NR. Score ≥6: sensitivity 89.52%, specificity 93.10%, PPV 98.23%, NPV 67.50%; accuracy 90.20%.	NR	DCA/utility: NR Explainability: No formal analysis	Scoring weights and thresholds in Table 1.	Clinical use or impact not evaluated	Laboratory values analyzed as associations are not inputs to the tested point score.

Study and reference	Discrimination AUC and threshold performance	Calibration	DCA or utility and explainability	Complete model specification availability	Clinical implementation or impact	Key notes and limitations
41 Huang et al. 2025 [53]	AUC training 0.774; holdout 0.863.	Calibration plots; MAE 0.035/0.050 (training/holdout)	DCA/utility: DCA reported in training and internal validation sets (Figure 8); clinical impact not established. Explainability: Tree/nomogram illustrated; no formal SHAP analysis	Graphical nomogram and liver-score equation available; complete final probability-model intercept/code NR.	Clinical use or impact not evaluated	Authors describe Calot-triangle clarity as preoperative US; acquisition/reproducibility requires scrutiny. Unknown imaging values are frequent. Main nomogram has seven predictors.
42 Suzuki et al. 2025 [7]	AUC development 0.788; internal holdout 0.769.	NR	DCA/utility: NR Explainability: No formal analysis	Seven-variable graphical nomogram and ORs provided; complete regression intercept/code NR.	No model-guided implementation evaluated; retrospective surgeon-expertise comparison	Prior EST is present in final nomogram although omitted from abstract predictor list. Serum CRP adds laboratory modality.
43 Zhu et al. 2025 [12]	AUC development 0.915 (95% CI 0.873–0.958); holdout 0.842 (0.737–0.947).	Calibration curves in both sets	DCA/utility: NR Explainability: No formal analysis	Nomogram/ORs and imaging definitions provided; complete intercept/coefficients/code NR.	Clinical use or impact not evaluated	Demographic/laboratory candidates were collected, but final model contains four imaging variables only.
44 Zhang et al. 2025 [6]	Development AUC 0.893 (95% CI 0.871–0.914); >3 identifies high risk. Later cohort: 15 direct open operations, 5 conversions and 11 complications.	NR	DCA/utility: NR Explainability: No formal analysis	Point weights and hierarchical management matrix complete in Tables 3–4.	Prospective score-guided allocation/open-surgery pathway; risk-group comparisons, without an untreated pathway control.	Implementation cohort does not establish a causal reduction in complications. Planned open surgery changes who remains eligible for conversion.
45 Wu et al. 2025 [13]	AUC development 0.868; external 0.833.	Calibration plots; HL P=0.9124/0.8537	DCA/utility: DCA; reported net-benefit ranges 1–81%/1–75% Explainability: No formal analysis	Coefficients/intercept in Table 2 and nomogram; exact categorical encoding requires checking for reimplementation.	Clinical use or impact not evaluated	TG18 coefficient is negative despite narrative risk direction. Stone-location/seniority coefficients are not statistically significant; source equation should be reproduced cautiously.
46 Naveed et al. 2025 [54]	AUC: NR. USS ≥5: sensitivity 73.8%, specificity 95.5%, PPV 79.4%, NPV 93.9%, accuracy 91.3%.	NR	DCA/utility: NR Explainability: No formal analysis	Complete thresholds and weights in Table I.	Clinical use or impact not evaluated	Evaluation uses ≥5; cited development paper used >5. Threshold transfer is therefore not identical.

Study and reference	Discrimination AUC and threshold performance	Calibration	DCA or utility and explainability	Complete model specification availability	Clinical implementation or impact	Key notes and limitations
47 Teja et al. 2025 [55]	AUC: NR. Reported conversion risk for scores 0/1/2: 0.5%/1.8%/7.2%; source denominators require caution.	NR	DCA/utility: NR Explainability: No formal analysis	Two one-point items reported; contracted-gallbladder measurement criterion insufficiently specified.	Clinical use or impact not evaluated	Percentages for score groups are not a formal calibration assessment. Overall conversion 9.5%; no independent validation.
48 Megahed et al. 2025 [56]	AUC 0.703; sensitivity 85.3%, specificity 88.5% reported. Preoperative/intraoperative correlation r=0.748.	NR	DCA/utility: NR Explainability: No formal analysis	Complete point table and risk bands in Table 2.	Clinical use or impact not evaluated	Threshold-dependent metrics and ROC AUC are not readily reconcilable; values retained. Inclusion wording for acute attacks/jaundice is unclear.
49 Ahmed et al. 2025 [57]	Preoperative AUC 0.948; reported sensitivity 95.5%, specificity 96.9%, PPV 94.1%, NPV 97.7%.	NR	DCA/utility: NR Explainability: No formal analysis	Preoperative weights in Table 2; 11 points sum, despite abstract describing 10.	Clinical use or impact not evaluated	Difficult-case counts conflict across abstract/Results/Table 5 (40%, 59/200, 67/200). AUC 0.94 for intraoperative score is a separate result.
50 Soniya et al. 2026 [58]	AUC: NR. Score >5: sensitivity 81.3%, specificity 88.2%, PPV 76.5%, NPV 90.9%; accuracy 86%.	NR	DCA/utility: NR Explainability: No formal analysis	Partial: seven variables and cutoff reported; individual point allocations not explicitly tabulated.	Clinical use or impact not evaluated	Wall/CBD boundary definitions differ between Methods and Results; four conversions (8%).
51 Moreno Delgado et al. 2026 [59]	Final AUC ≈0.89; CV mean 0.861 (SD 0.094); bootstrap 0.882 (95% CI 0.829–0.904). Score ≥7: sensitivity 77.8%, specificity 85.9%.	NR	DCA/utility: NR Explainability: No formal analysis	Point score complete in Table 1: weights 2/2/3/3/3/2; probability-model intercept NR.	Clinical use or impact not evaluated	SII added after review of misclassified cases. Cohort includes age 15. Published 8 January 2026; acceptance/copyright dated 2025.
52 Sudhir et al. 2026 [60]	AUC 0.835 (95% CI 0.724–0.947). Optimal cutoff 9: 100% sensitivity/specificity reported.	NR	DCA/utility: NR Explainability: No formal analysis	Point allocations and risk bands provided in Table 1.	Clinical use or impact not evaluated	Perfect classification at cutoff 9 is not readily consistent with reported AUC; source values retained. Thirteen conversions (21.7%).
53 Yang et al. 2026 [14]	NB AUC: development 0.881 (95% CI 0.848–0.914); external 0.868 (0.810–0.921).	Calibration curves in development and external cohorts	DCA/utility: DCA in both cohorts Explainability: SHAP	Online calculator reported; complete trained NB parameters/code NR in main text.	Calculator developed; no evaluated care/impact study	Pooled multicenter recruitment is separated from genuine hospital-based external validation. Outcome and some variable wording require careful interpretation.
54 Şenocak et al. 2026 [61]	AUC HCRM/CCRM/TG18: 0.927/0.931/0.576; corrected 0.912/0.903/0.580. Point score AUC 0.893; +TG18 0.891.	Curves/slopes and HL tests; HCRM slope 1.11, CCRM 1.13. Brier also reported.	DCA/utility: DCA reported for regression-model comparison Explainability: No formal analysis	Point increments and cap complete in Table 5. Regression coefficients shown; intercept/full probability equation NR in main text.	Clinical use or impact not evaluated	Calibration and DCA are reported, contrary to previous charting. Point score Brier 0.082. TG18 adds little discrimination; no external validation.

Study and reference	Discrimination<br>AUC and threshold performance	Calibration	DCA or utility and explainability	Complete model specification availability	Clinical implementation or impact	Key notes and limitations
55 Basukala et al.<br>2026 [62]	AUC 0.890 (95% CI 0.853–0.926). Cutoff 6.5: sensitivity 89.1%, specificity 21.0%, as reported.	Score: NR.<br>Secondary regression: Hosmer–Lemeshow P=0.249.	DCA/utility: NR<br>Explainability: No formal analysis	Scoring form and multivariable results cited as online supplements; not present in uploaded PDF.	Clinical use or impact not evaluated	Low reported specificity contrasts with high AUC; source values retained. Age 17 included. Do not attribute secondary-model calibration to the score.
56 Kumar et al.<br>2026 [63]	AUC 0.749 (95% CI 0.672–0.816). Score >3: sensitivity 57.14%, specificity 79.51%, PPV 39%, NPV 85% (Results).	NR	DCA/utility: NR<br>Explainability: No formal analysis	Table 1 provides point rule; hospitalization assigned 1 point (original rule uses 4).	Clinical use or impact not evaluated	NPV differs between abstract and Results. Secondary regression includes intraoperative variables and is not a preoperative prediction model.
57 Nagarla et al.<br>2026 [64]	AUC 0.927. Score >6: sensitivity 75.76%, specificity 98.29%; LR+ 44.3.	NR	DCA/utility: NR<br>Explainability: No formal analysis	Partial: point table provided; elevated-ALP cutoff and some exact boundaries NR.	Clinical use or impact not evaluated	Acute cholecystitis is excluded but also appears as a scoring item. Duplicate unnumbered PDF contains the same paper.
58 Ayantunde et al.<br>2026 [65]	AUC 0.797 (95% CI 0.742–0.851). Threshold ≥6: sensitivity 39.3%, specificity 97.0%, PPV 87.3%, NPV 75.2%.	NR	DCA/utility: NR<br>Explainability: No formal analysis	Risk bands 0–3/4–6/≥7 described; complete weighted form cited in Online Appendix 1, not supplied.	Used at theatre booking; 90/120/180-min allocation and surgeon review. No controlled impact comparison.	Observed workflow use is documented; outcome correlations do not demonstrate a causal improvement in care.
59 Utsumi et al.<br>2026 [15]	AUC development 0.806 (95% CI 0.747–0.866); holdout 0.760 (0.654–0.865). At probability 0.40: holdout sensitivity 72%, specificity 69%.	Bootstrap-corrected curves; mean absolute errors 0.015/0.028	DCA/utility: DCA in both sets<br>Explainability: No formal analysis	Nomogram and predictor definitions shown; complete coefficients/intercept/code NR.	Clinical use or impact not evaluated	Calibration-plot sample sizes 211/82 differ from nominal 223/88. Outcome boundary differs between abstract and Methods; no external validation.

*ADS, adhesions detection and staging; AI, artificial intelligence; ALP, alkaline phosphatase; ALT, alanine aminotransferase; ARFI, acoustic radiation force impulse; ASA, American Society of Anesthesiologists physical status; AST, aspartate aminotransferase; AUC/AUROC, area under the receiver operating characteristic curve; BMI, body mass index; CAR, C-reactive protein to albumin ratio; CCRM, clinical conversion risk model; CBD, common bile duct; CI, confidence interval; CLOC, conversion from laparoscopic to open cholecystectomy; CPM, Cairns Prediction Model; CRP, C-reactive protein; CRS, conversion risk score; CSS, clinical scoring system; CT, computed tomography; CV, cross-validation; CVS, critical view of safety; DCA, decision-curve analysis; DiLC/DLC, difficult laparoscopic cholecystectomy; ED, emergency department; ERCP, endoscopic retrograde cholangiopancreatography; EST, endoscopic sphincterotomy; GB, gallbladder; GPS, Glasgow Prognostic Score; HCRM, hybrid conversion risk model; HL, Hosmer–Lemeshow; LASSO, least absolute shrinkage and selection operator; LC, laparoscopic cholecystectomy; LDH, lactate dehydrogenase; LDL, low-density lipoprotein; LOS, length of stay; LR, likelihood ratio; LVS, Labbad–Vivas score; MAE, mean absolute error; ML, machine learning; MRCP, magnetic resonance cholangiopancreatography; NB, naive Bayes; NLR, neutrophil-to-lymphocyte ratio; NPV, negative predictive value; NR, not reported; PGS, Parkland Grading Scale; PLR, platelet-to-lymphocyte ratio; POS, preoperative score; PPV, positive predictive value; PTGBD, percutaneous transhepatic gallbladder drainage; RF, random forest; RSCLO, risk score for conversion from laparoscopic to open cholecystectomy; SD, standard deviation; SE, standard error; SHAP, SHapley Additive exPlanations; SII, systemic immune-inflammation index; SVM, support vector machine; TBIL, total bilirubin; TG18, Tokyo Guidelines 2018; US/USS, ultrasonography; VTI, virtual touch tissue imaging; WBC, white blood cell count; n, number of patients.

Panel D Machine learning development procedures and reporting

Study	Class imbalance handling	Feature selection	Hyperparameter tuning	Cross-validation	Data leakage prevention
**Sun et al., 2025 [10]**	SMOTE reported; restriction to training data/folds unclear.	mRMR and LASSO for radiomics; Boruta–LASSO for clinical predictors.	LASSO λ selected by cross-validation; classifier-specific tuning NR.	10-fold CV; 70:30 training/holdout split.	Training-only preprocessing, feature selection, and resampling procedures not explicitly documented.
**Agrawal et al., 2025 [52]**	NR.	Preoperative scores linked to operative-derived scores through co-occurrence matrices; reproducible feature-selection rule NR.	NR.	NR; first 17 patients used for training and next eight for testing.	Separate training/test groups described; safeguards for deriving and applying score mappings insufficiently specified.
**Huang et al., 2025 [53]**	NR.	LASSO, SVM-based variable selection, decision-tree and logistic-regression analyses.	LASSO λ selection illustrated using AUC; SVM/tree tuning details NR.	CV reported for SVM variable selection; number of folds NR. Bootstrap resampling also reported.	Training/holdout split described; restriction of variable selection to training data and CV folds unclear.
**Yang et al., 2026 [14]**	NR.	LASSO followed by multivariable logistic regression and correlation filtering.	LASSO λ selected by 10-fold CV; classifier-specific tuning NR.	10-fold CV in training data; validation using two other hospitals.	Hospital-based separation described; training-only standardization and feature selection, including nesting within CV, not explicitly documented.

*NR = not reported in the assessed publication. Missing methodological details were not inferred. A reported training/validation split or cross-validation procedure was not considered sufficient evidence that all preprocessing, feature selection, resampling, and tuning steps were performed without data leakage.
