# Supplementary material for: Preoperative Prediction of Difficult Laparoscopic Cholecystectomy: Evolution, Validation, and Emerging Machine-Learning Approaches—A Scoping Review
Source: Medicina (Kaunas). 2026 Oct 21;62(10):2042. doi: 10.3390/medicina62102042 (PMC13707204; doi:10.3390/medicina62102042)
Supplement: Supplementary file 1 [file medicina-62-02042-s001.zip › Supplementary_Table_S2.pdf]

Supplementary Table S2. Full-text retrieval and eligibility audit

Reports not retrieved and full-text exclusion from the final database-derived evidence set

Of 64 reports sought for retrieval, four could not be obtained through the prespecified accessible sources. The remaining 60 reports underwent full-text eligibility assessment; one was excluded because the final prediction model required postoperative histopathological information.

**[67]**	Martínez-Mier G, Méndez-Rico D, Reyes-Ruiz JM, Moreno-Ley PI, Bernal-Dolores V, Avila-Mercado O. External Validation of Two Scoring Tools to Predict the Operative Duration and Open Conversion of Elective Laparoscopic Cholecystectomy in a Mexican Population. Digestive Surgery. 2023;40(3-4):108-113. doi:10.1159/000531087.	**Report not retrieved**	Full text could not be retrieved through the prespecified accessible sources.	The report did not undergo full-text eligibility assessment. Study authors were not contacted, in accordance with the registered protocol.
**[68]**	Ibrahim Y, Radwan RW, Abdullah AAN, Sherif M, Khalid U, Ansell J, Rasheed A. A Retrospective and Prospective Study to Develop a Pre-operative Difficulty Score for Laparoscopic Cholecystectomy. Journal of Gastrointestinal Surgery. 2019;23(4):690-695. doi:10.1007/s11605-018-3821-y.	**Report not retrieved**	Full text could not be retrieved through the prespecified accessible sources.	The report did not undergo full-text eligibility assessment. Study authors were not contacted, in accordance with the registered protocol.
**[69]**	Kim MS, Kwon HJ, Park HW, Park JY, Chung EC, Park HJ, Kwag HJ, Hong HP. Preoperative Prediction Model for Conversion of Laparoscopic to Open Cholecystectomy in Patient With Acute Cholecystitis: Based on Clinical, Laboratory, and CT Parameters. Journal of Computer Assisted Tomography. 2014;38(5):727-732. doi:10.1097/RCT.0000000000000116.	**Report not retrieved**	Full text could not be retrieved through the prespecified accessible sources.	The report did not undergo full-text eligibility assessment. Study authors were not contacted, in accordance with the registered protocol.
**[70]**	Wevers KP, van Westreenen HL, Patijn GA. Laparoscopic Cholecystectomy in Acute Cholecystitis: C-Reactive Protein Level Combined With Age Predicts Conversion. Surgical Laparoscopy, Endoscopy & Percutaneous Techniques. 2013;23(2):163-166. doi:10.1097/SLE.0b013e31826d7fb0.	**Report not retrieved**	Full text could not be retrieved through the prespecified accessible sources.	The report did not undergo full-text eligibility assessment. Study authors were not contacted, in accordance with the registered protocol.
**[71]**	Stanisic V, Milicevic M, Kocev N, Stanisic B. A prospective cohort study for prediction of difficult laparoscopic cholecystectomy. Annals of Medicine and Surgery. 2020;60:728-733. doi:10.1016/j.amsu.2020.11.082.	**Full-text report excluded**	Prediction model required postoperative histopathological information.	The final model included gallbladder fibrosis derived from pathohistological examination of the resected gallbladder. Because this predictor is unavailable before surgery, the model did not meet the prespecified preoperative-only eligibility criterion.

Notes. Reports classified as “not retrieved” were not assessed for full-text eligibility and were not counted as full-text exclusions. In accordance with the registered protocol, study authors were not contacted to obtain unavailable reports. One primary exclusion reason was assigned to the single retrieved report excluded after full-text assessment. “Preoperative-only” denotes that every input required to calculate the final prediction tool had to be available before the start of surgery.

Final audit totals: reports sought for retrieval, n=64; reports not retrieved, n=4; reports assessed for eligibility, n=60; full-text reports excluded, n=1; studies included in the final review, n=59.
